# Abscopal Effect and Drug-Induced Xenogenization: A Strategic Alliance in Cancer Treatment?

**DOI:** 10.3390/ijms221910672

**Published:** 2021-10-01

**Authors:** Ornella Franzese, Francesco Torino, Elisa Giannetti, Giorgia Cioccoloni, Angelo Aquino, Isabella Faraoni, Maria Pia Fuggetta, Liana De Vecchis, Anna Giuliani, Bernd Kaina, Enzo Bonmassar

**Affiliations:** 1 School of Medicine, Department of Systems Medicine, University of Rome Tor Vergata, 00133 Rome, Italy; franzese@uniroma2.it (O.F.); g.cioccoloni@leeds.ac.uk (G.C.); angelo.aquino@uniroma2.it (A.A.); faraoni@med.uniroma2.it (I.F.); lianadev49@yahoo.com (L.D.V.); 2 Department of Systems Medicine, Medical Oncology, University of Rome Tor Vergata, 00133 Rome, Italy; torino@med.uniroma2.it (F.T.); elisag1611@gmail.com (E.G.); 3 School of Food Science and Nutrition, University of Leeds, Leeds LS29JT, UK; 4 Institute of Translational Pharmacology, Consiglio Nazionale delle Ricerche (CNR), Via Fosso del Cavaliere, 00133 Rome, Italy; mariapia.fuggetta@ift.cnr.it (M.P.F.); agiuliani37@yahoo.it (A.G.); 5 Institute of Toxicology, University Medical Center, D-55131 Mainz, Germany

**Keywords:** abscopal effect, xenogenization, alkylating agents, dacarbazine, temozolomide, immune response, immune checkpoints, tumor neoantigens

## Abstract

The current state of cancer treatment is still far from being satisfactory considering the strong impairment of patients’ quality of life and the high lethality of malignant diseases. Therefore, it is critical for innovative approaches to be tested in the near future. In view of the crucial role that is played by tumor immunity, the present review provides essential information on the immune-mediated effects potentially generated by the interplay between ionizing radiation and cytotoxic antitumor agents when interacting with target malignant cells. Therefore, the radiation-dependent abscopal effect (i.e., a biological effect of ionizing radiation that occurs outside the irradiated field), the influence of cancer chemotherapy on the antigenic pattern of target neoplastic cells, and the immunogenic cell death (ICD) caused by anticancer agents are the main topics of this presentation. It is widely accepted that tumor immunity plays a fundamental role in generating an abscopal effect and that anticancer drugs can profoundly influence not only the host immune responses, but also the immunogenic pattern of malignant cells. Remarkably, several anticancer drugs impact both the abscopal effect and ICD. In addition, certain classes of anticancer agents are able to amplify already expressed tumor-associated antigens (TAA). More importantly, other drugs, especially triazenes, induce the appearance of new tumor neoantigens (TNA), a phenomenon that we termed drug-induced xenogenization (DIX). The adoption of the abscopal effect is proposed as a potential therapeutic modality when properly applied concomitantly with drug-induced increase in tumor cell immunogenicity and ICD. Although little to no preclinical or clinical studies are presently available on this subject, we discuss this issue in terms of potential mechanisms and therapeutic benefits. Upcoming investigations are aimed at evaluating how chemical anticancer drugs, radiation, and immunotherapies are interacting and cooperate in evoking the abscopal effect, tumor xenogenization and ICD, paving the way for new and possibly successful approaches in cancer therapy.

## 1. Introduction 

In the last 30 years, patients affected by most malignancies have obtained substantial advantages in terms of treatment by early diagnosis and combined anticancer approaches. However, survival remains unsatisfactory in many advanced solid malignancies (e.g., lung, gastric, colorectal, pancreatic, urological, brain tumors, and melanoma). Besides the classical cytotoxic chemotherapy, targeted therapy has gained an attractive area of intervention with almost daily novel biological approaches [1]. Recent progresses and future promises of synthetic lethality and targeted therapy (including antibody-drug conjugates) against cancer stem cells [2,3,4,5] could foresee attractive perspectives, although in almost all cases the onset of chemo-resistance appears to be inevitable, thus compromising the therapeutic efficacy after an initial period of tumor regression or control. 

Among anticancer approaches, a fundamental role is played by radiotherapy (RT) for loco-regional control of several neoplastic diseases; however, in disseminated malignancies RT alone has not been considered for therapy because of toxic [6] and severe immunosuppressant effects [7]. However, stereotactic RT for oligometastatic tumors has been utilized in clinical studies (for a review, see [8]), with acceptable toxicity and potential benefit also in terms of overall survival (OS) [9]. 

Hormone therapy plays a well-established role since it can be considered a treatment with limited side effects and capable of inducing long-term disease-free intervals in hormone-sensitive tumors. In the adjuvant setting for the post-surgery control of susceptible tumors, hormone therapy is considered a mainstay treatment, either alone or in combination with chemotherapy, although the long-term treatment with this therapeutic approach is not devoid of limited but clear side effects. In advanced malignancies, endocrine therapy has a more limited role and shows often non-durable cytostatic effects.

Most encouraging is the consolidated field of cancer immunotherapy [10,11], which is directed against essentially two types of tumor antigens, which we refer to as “tumor-associated antigens” (TAA) and “tumor neoantigens” (TNA). All tumor antigens that can also be detected in normal mature or embryonic cells, but are more expressed in malignant cells and susceptible to tolerance breakage, will be considered TAA. All tumor antigens exclusively present in tumor cells, induced by oncogenic processes or by physical or chemical agents, will be designated as TNA. It must be underlined that immuno-mediated tumor killing elicited by immune checkpoint inhibitors (ICIs) [12,13,14]), including most recent agents of clinical interest [15], has led to substantially extension of survival in an increasing number of malignancies, being even curative in selected conditions [12]. However, resistance to ICIs can be observed in up to 70% of patients [14], and ICI-induced autoimmunity may be a major clinical concern [13]. 

Starting with the current scenario, it is reasonable to consider that the future development of cancer treatment should be based on the rational alliance among different therapeutic modalities, dictated by the molecular profile of the malignancy affecting a particular patient.

In this review, we discuss the potential contribution to cancer therapy of a novel approach derived from the combination of chemotherapy, immunotherapy, and RT within the context of the “abscopal effect.” The term, derived from “ab scopus” (meaning “outside the target”), was introduced by R.H. Mole in 1953 [6] to describe a biological effect mediated by ionizing radiation, which occurs outside the irradiated field. A number of preclinical and clinical studies showed that in tumor-bearing hosts subjected to RT, regression of non-irradiated neoplastic lesions (in most cases metastases) can occasionally occur on sites distant from those exposed to ionizing radiation (reviewed in [16,17] and for a historical review [18]). It is reasonable to predict that treatments aimed at amplifying this phenomenon could have a considerable impact on cancer management, especially in the metastatic and disseminated stage of the disease.

**Figure 1:**
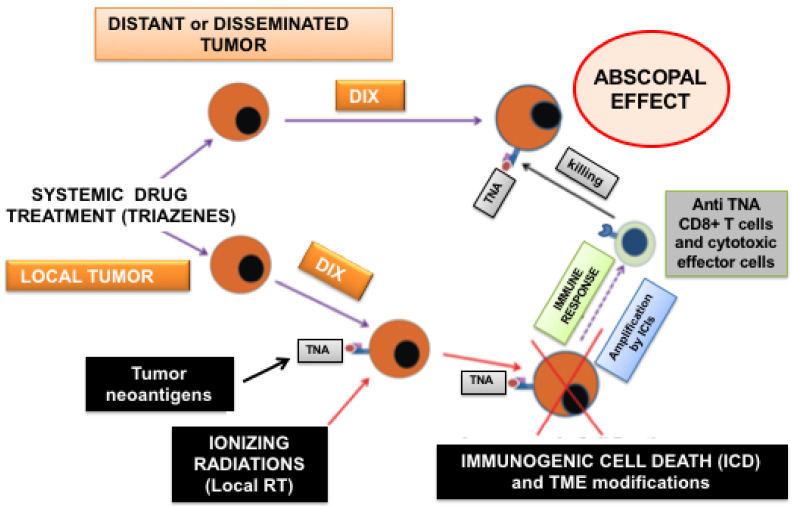
Main players involved in the chemo-radiation-induced and immuno-mediated abscopal effect. Systemic administration of selected drugs (e.g., triazene compounds) in tumor-bearing hosts can result in the induction of novel tumor antigens (tumor neoantigens, TNA, presented by MHC molecules), a phenomenon that has been termed “drug-induced xenogenization” (DIX, [19]). A portion of the malignant cells (e.g., a metastatic lesion in a clinical setting of a local “in situ vaccine” for advanced solid tumors [20]) is exposed to ionizing radiation. RT must be properly delivered to avoid immune suppression and to produce immunogenic cell death (ICD) and tumor microenvironment (TME) modifications that are able to activate systemic cell-mediated immunity. Immunotherapy (IT) including immune checkpoint inhibitors (ICIs) also plays an important role in generating cytotoxic effector cells against non-irradiated distant and/or disseminated *xenogenized* tumor cells (abscopal effect).

As illustrated in Figure 1, the efficacy of the abscopal effect depends on four essential factors: (1) tumor cell immunogenicity, (2) efficient ICD, (3) a tumor microenvironment (TME) favoring immuno-mediated response, and (4) suppression of tumor cell defenses against a host’s immune attack. Crucial factors playing a role of primary importance are obviously the technique and modality of radiation delivery. However, we will not deal with this parameter in depth, since it is highly technically oriented and is outside the scope of this review. Particular attention will be dedicated to tumor cell immunogenicity since treatment with selected drugs, belonging notably to the class of DNA methylating agents, is able to induce highly immunogenic neoantigens in cancer cells (i.e., the DIX phenomenon [19]), thus potentially intensifying the RT-mediated abscopal effect.

## 2. Abscopal Effect

Local treatment of malignant cells with various physical and chemical agents can be followed by systemic outcomes that can be classified as abscopal effects. Here, we focus on the RT-induced abscopal effect. Preclinical and clinical investigations have produced convincing data that the immune system plays a significant role in this process, although other non-immunological mechanisms could be involved. Actually, Tesei et al. [21] showed that irradiated lung tumor cells secrete vesicles expressing p53-dependent pro-senescence molecules capable of inhibiting the growth of non-irradiated “distant” malignant cells. Moreover, radiation has been shown to increase membrane-bound levels of ceramide by stimulating acid sphingomyelinase (ASMase), which is able to release ceramide from sphingomyelin [22], inducing wide lipid raft alterations, mainly implicated in cell apoptotic processes [23,24] and in endothelial damage, ultimately emerging in vascular deterioration [25,26]. 

### 2.1. Preclinical and Clinical Investigations

The abscopal effect has been reported for different mouse models and irradiated targets such as breast [27,28], lung [28], colorectal [20], and pancreatic [29] cancer and malignant melanoma [30]. A meta-analysis of the literature concerning dose and modality of ionizing radiation delivery in preclinical models conducted by Marconi et al. [31] illustrated the best conditions of stereotactic ablative radiotherapy (SABR) required to obtain a reliable abscopal effect. Moreover, Brooks and Change pointed out that higher probabilities of successful therapeutic outcomes could be obtained if more than one neoplastic site were subject to appropriate RT [32].

Based on this groundwork and on the observation that the abscopal effect relies on local RT-induced amplification of anti-tumor immunity (see below), many clinical studies have been conducted with RT prevalently of the SABR type, often in combination with immune checkpoint inhibitors (ICIs). Results obtained in case studies or in more structured clinical investigations are compiled in Supplementary Material Table S1. However, as stated by Zhuang [33], the precise role played by the abscopal effect either alone or associated with ICIs has not been conclusively assessed in cancer therapy, since large-scale randomized studies with definite parameters are presently not available. 

The need for further sizable research efforts is dictated by the finding that in several cases, this therapeutic design might not be able to guarantee long-lasting responses due to limited tumor immunogenicity or to the ability of malignant cells to mount successful strategies of escaping from immune surveillance (see below). Moreover, critical questions remain open in defining optimal dosing and treatment schedules concerning RT either alone (as recently highlighted by Demaria et al. [34]) or in association with various immunotherapy approaches [35,36].

### 2.2. Mechanism of RT-Induced Abscopal Effect and Immunogenic Consequences of Radiation

A deep understanding of the mechanism underlying the abscopal effect is needed to identify the associated therapeutic targets and exploit this phenomenon as an efficient therapeutic tool. An exhaustive update of our knowledge on this subject has been provided by Rodriguez-Ruiz [37] and by Lhuillier et al. [38]. It is evident that local RT is able to generate chemical and/or cell-mediated responses, producing factors that diffuse to distant sites where they provoke biological effects. 

Either spontaneous or drug- or radiation-induced somatic mutations, frequently occurring in malignant cells, could be responsible for the appearance of TNA that can be recognized by T cells. In particular, by generating point mutations producing immunogenic tumor-associated peptides, RT can potentially amplify the cross T-cell responses to the native antigens expressed by non-irradiated tumor cells. This possible scenario is reminiscent of the observation that mice sensitized against syngeneic lymphomas that are highly immunogenic by drug-induced mutagenesis are also able to reject weakly immunogenic parental lymphoma cells [39]. Actually, RT also appears to amplify the immunogenicity of TNA through an increase in their expression, which is frequently accompanied by MHC upregulation on cancer cells [40] and a more efficient recognition by the host’s T cells, thus producing a sort of in situ tumor vaccine. 

The immuno-mediated mechanism responsible for the abscopal effect has been at least in part revealed by several studies showing that RT is able to induce a “viral mimicry” effect in target cells consisting mainly of the induction of the type 1 interferon and its release in the TME [41]. Damage of nuclear and mitochondrial [42] DNA produced by RT generates fragments of double-stranded DNA (dsDNA) that migrate into the cytoplasm and activate DNA-sensing molecules [43]. In particular, the presence of dsDNA is sensed by cyclic GMP-AMP synthase (cGAS) that interacts with dsDNA, leading to the formation of a cGAS-dsDNA dimer [41]. Thereafter, the dimer recognizes ATP and GTP substrates, generating the cyclic dinucleotide 2’,3’-cyclic GMP-AMP (cGAMP) complex. In turn, in the endoplasmic reticulum (ER), cGAMP activates the stimulator of interferon genes (STING) that undergoes tetramerization and subsequent palmitoylation [44]. Palmitoylated STING interacts with TANK-binding Kinase 1 (TBK1) [45] and phosphorylates the interferon regulatory factor 3 (IRF3). After phosphorylation, IRF3 dimerizes, translocates to the nucleus, and activates type 1 interferon genes [46,47].

Moreover, ionizing radiation can activate endogenous retrovirus transcription [48], generating double-stranded RNA (dsRNA) that is shuttled to the cytoplasm [49]. Cytoplasmic dsRNA is sensed by the pattern-recognition receptor retinoic acid-inducible gene-1 (RIG-1) and by the RIG-1-like receptor melanoma differentiation-associated protein 5 (MDA5) [50]. Both RIG-1 and MDA5 after dsRNA binding activate the mitochondrial antiviral-signaling protein (MAVS) at the mitochondrial level. MAVS activates the TNF receptor associated factor (TRAF), which in turn activates two kinases (i.e., TBK1 and IKKs [51]) that phosphorylate IRF3 and IRF7. Thereafter, following dimerization, both factors migrate into the nucleus and activate the transcription of the IFN gene. In conclusion, both RNA and DNA short-size double-stranded nucleotide chains interact with cytoplasmic sensors and trigger the signaling pathways, leading to an increase in type 1 IFN expression [52]. 

An essential role is played by the GAS/STING system that is involved in the production of cytokines, impacting both the innate and the adaptive antitumor immunity [43,53,54,55], similarly to the events elicited by viral infections, i.e., the abovementioned “viral mimicry” [47,56]. This model of the cGAS-STING pathway describes the type I interferon response as a consequence of cytosolic DNA. However, the relationship between irradiation, DNA damage, and immune response can also be explained on the basis of investigations that revealed how DNA sense might occur in cGAS-containing micronuclei created through abnormal mitosis following DNA damage [57,58]. In addition, while several studies indicate a tumor cell-based activation of STING [59,60], others have described how exosomes containing DNA molecules are released and are potentially able to stimulate STING signaling in trans, fueling the radiation-related immune response [61,62]. 

The immune cell features within the TME are strongly influenced by the cytokine composition, which is highly impacted by RT. The pro-inflammatory conversion occurring in the TME after irradiation contributes to the engagement of lymphocytes and the improvement in effector T-cell activation [63] 

One of the main final outcomes of the described induction of interferon release is the activation of a subset of Th1 response associated CD11c+, CD8α+ BATF+ dendritic cells (DCs) [64] that migrate to lymph nodes, where they provoke cross-priming of effector CD8+ T cells that will access either the principal lesion or the non-irradiated distant sites, contributing to tumor eradication [65].

In pre-clinical models, radiation has also been shown to stimulate the production of T helper 1 (Th1)-related chemokine CXC-chemokine ligand 10 (CXCL10) [66], which is strongly involved in tumor infiltration by CD8+ effector T cells through engagement to CXCR3 [67]. The abovementioned CD11c+CD8α+ BATF+ DCs, induced by radiation, have also been described to play a critical role in CXCL10 as well as CXCL9 production [64]. 

However, whereas low-dose radiation has been mainly related to a Th1-like response in the TME, characterized by the secretion of IFN-γ and TNF-α [68], a Th1/Th2 polarization has also been reported in association primarily with high doses [69]. Nevertheless, a single radiation dose of 15 Gy has been shown to improve DC maturation in the B16 melanoma model [70], whereas high single doses (> 20 Gy) significantly improve the priming of CD8+ T cells, intra-tumor T cell infiltration, and tumor regression in preclinical models of murine tumors [71,72].

Another aspect of positive immunomodulating activities of RT concerns the dose-dependent upregulation of MHC-I expression on tumor cells, thus favoring tumor antigen presentation to T cells. In particular, better MHC induction was obtained with single doses >4 and 8 up to 20 Gy in melanoma and colon cancer, respectively [73]. 

Remarkably, irradiation alone has also been shown to broaden tumor-specific T-cell repertoire (TCR), probably as a consequence of neo-antigen burden widening, but is also able to stimulate the expansion of ICI-induced oligoclonal anti-tumor T-cell clones when used in combination with a CTLA-4 blockade [74]. This provides additional evidence of a potential cooperation between immune therapy and RT.

### 2.3. RT-Induced Immunosuppression 

The mechanisms underlying RT-associated immune modulation have been exhaustively described in recent studies (reviewed in [75]) and include, as previously highlighted, immuno-stimulating pathways involved in the immuno-mediated abscopal effect. 

However, exposure to RT triggers various immunosuppressive mechanisms, including the recruitment of regulatory T (Treg) cells [76], M2 macrophages, and myeloid-derived suppressor cells (MDSCs) [36]. Moreover, RT contributes to the generation and activation of cancer-associated fibroblasts that provide immunosuppressive cytokine signaling [77]. Interestingly, RT also causes upregulation of PD-L1, which was shown to be mediated by the hypoxia-inducible factor-1α (HIF-1α) [37]. Since the membrane-localized PD-L1 on cancer cells contributes to tumor evasion from the immunological control, it is reasonable to conclude that RT-induced PD-L1 upregulation negatively impacts curative radiation responses, including attenuating the abscopal effect [37,78,79,80,81]. This finding provides a rational basis for introducing immunotherapy with anti-PD-1/PD-L1 monoclonal antibodies along with RT.

Remarkably, unlike monocytes, macrophages, and DCs, a high sensitivity of T lymphocytes to RT has been demonstrated, with low doses sufficient to induce DNA damage and apoptosis [82], which is supposed to cause transient T-cell depletion in the TME.

In this context, the purinergic CD39/CD73/adenosine system, already described in 2007 [83], has recently caught the attention of immunologists as a critical endogenous regulator of the innate and adaptive immune systems with a documented role in tumor immune escape. The importance of the negative influence of the purinergic system on triggering the abscopal effect, and in general on RT, stems from the finding that ionizing radiation substantially activates the CD73/adenosine signaling pathway [84]. Both CD73 and CD39 are 5′ectonucleotidases mainly produced by specific T-cell subsets, including Tregs and several T-cell subgroups as well as stromal and tumor-associated stem cells [85]. The biological function of these enzymes concerns extracellular ATP metabolism since CD39 converts ATP into ADP and AMP, whereas CD73 converts AMP into adenosine [86]. In turn, adenosine interacts with specific purinergic receptors [87] and provides immunodepressive signaling frequently exploited by tumors to evade immune surveillance. Actually, adenosine possesses pleiotropic immunosuppressive effects and reduces activation of DC and effector T cells while promoting the activity of Tregs [88,89,90]. Therefore, the purinergic CD39/CD73/adenosine system contributes to shaping different aspects of a tumor-driven immune scenario in the TME. This is particularly evident in hypoxic cancers [91,92] where effector T-cell function is impaired mainly through the binding of adenosine to the A2A receptor expressed by CD8+T cells [91]. 

Exposure of human and mouse breast cancer cells to irradiation is followed by increased expression of CD73 [93,94], which also plays a critical role in promoting radiation-induced toxicity, chronic inflammation, and fibrosis in normal lung tissue [95]. Presently, all available preclinical data emphasize the worth of CD73 as a potential therapeutic target for cancer control [96,97], encouraging clinical studies aimed at evaluating the use of anti-CD73 MEDI9447 mAb and/or LY3475070 small inhibitor molecules alone or in combination with a PD-1 blockade in patients with advanced solid tumors [98]. Moreover, it is conceivable that CD73 suppression can represent a favorable approach able to increase cooperation between RT and immunotherapy. Indeed, blockade of CD73 has been shown to enhance radiation-associated tumor infiltration by DC in the absence of type 1 IFN induction while improving systemic antitumor T-cell responses [94]. In the LuM-1 preclinical model, a highly metastatic murine colon cancer, expression of CD73 was significantly increased after RT [99]. In subcutaneous lesions of the same tumor model and microscopic pulmonary metastases occurring in Balb/c mice, characterized by increased levels of adenosine after RT, intraperitoneal anti-CD73 antibody alone did not produce antitumor effects. In contrast, when combined with RT, anti-CD73 treatment delayed tumor growth while suppressing the development of lung metastases, presumably through abscopal effects, leading to enhanced anti-tumor cytotoxicity compared to controls. However, the most favorable approach to be employed for targeting CD73 in combination with RT has not been identified yet, in terms of either whole dose and quality of radiation, or in the sequence of treatment [100,101,102]. Indeed, optimal treatment conditions must be determined, taking into account the impact of RT and the activation of CD73/adenosine signaling in both tumor and normal tissues, where excessive inflammation or autoimmunity may develop by inhibiting CD73-adenosine protective signals.

Remarkably, different radiation doses and dose-fractionation have been associated with a distinctive degree of modulation of immune responses. In particular, while classical portioning has been associated with an increased frequency of MDSCs and Treg cells in the TME [103,104], low-dose RT generally promotes more immunogenic changes in the TME, including the polarization of TAMs to an M1 phenotype [105] and a Th1 cytokine setting [106]. Accordingly, in a study involving 47 metastatic melanoma patients treated with ipilimumab and RT, the abscopal effect emerged as being more successful with a reasonably hypo-fractionated dose inferior to 3 Gy compared to a dose higher than 3 Gy, which impaired the abscopal effect by abolishing cell-mediated immune responses [107]. In conclusion, all these observations suggest that radiation dosage and partition, along with the distinctive TME features, contribute to explaining the double-edged sword represented by RT in terms of immunoenhancement or immunosuppression [108]. More in-depth individual analyses are needed, together with reliable patient response markers, to identify how irradiation in combination with ICIs can be optimally turned into a therapeutic advantage [36,109,110], leaving open the option of a purinergic pathway blockade. 

## 3. Drug-Induced Upregulation of Tumor Immunogenicity

Tumor antigen(s) that can be recognized by tumor-bearing hosts provide the immunological bases of the abscopal effect. Therefore, a critical role is played by the level of malignant cell immunogenicity. As stated in the introduction, two categories of tumor antigens must be taken into consideration, i.e., TAA, which is preferentially present but not solely expressed by neoplastic cells (e.g., epitopes of MUC1, HER2/neu, or CEA origin [111]), and TNA, which is exclusively expressed by malignant cells [112]. It follows that the level of cross-reacting TAA or TNA presented either by the locally irradiated tumor or distant metastatic lesions appears to be of great relevance for the induction of the abscopal effect. Therefore, we will consider some pharmacological strategies that could be adopted to increase TAA expression or to induce TNA appearance. These effects can be defined as drug-induced antigen remodeling, and refer to any pharmacological stimulus able to modify the antigenic pattern of malignant cells in the course of tumor growth. In particular, certain drugs could increase the expression of pre-existing TAA (e.g., 5-fluorouracil [113], see below) and others could be able to provoke the de novo generation of TNA (e.g., genotoxic antitumor agents [19,114,115]). The presence of neoantigens may also result from “spontaneous” tumor cell mutations responsible for drug resistance [116,117]. However, it should be stressed that antigen remodeling must not necessarily lead to favorable outcomes. It cannot be excluded that treatment with certain drugs might also restrain the immunogenic properties of cancer cells by reducing TAA or TNA expression or their presentation on cancer cells [118] or by increasing the intra-tumor heterogeneity as a consequence of accumulating mutations in a subset of cancer cells [119,120].

It is important to consider that in most cases classical antitumor drugs (e.g., anthracyclines, platinum compounds, cyclophosphamide, bortezomid, etc.) are able to induce ICD (see next section) rather than causing upregulation of TAA or TNA expression in tumor cells [121].

Moreover, besides their influence on tumor-cell antigenic patterns, cytotoxic anticancer drugs can provide remarkable pharmacodynamic effects on host’s immune responses through selective killing of specific immunocompetent cell populations. An example is provided by cyclophosphamide, which stimulates the immune response if administered at low (non-toxic) dose levels [122,123]. This was attributed to selective cytotoxicity on CD4+CD25+ Tregs, which turned out to be more sensitive to cyclophosphamide than cytotoxic T cells (Tc) and Th cells, presumably due to defective repair of cyclophosphamide-induced DNA crosslinks [124]. The hypersensitivity of Tregs is restricted to cyclophosphamide and does not pertain to other alkylating drugs like temozolomide. It results in a decline in the suppressor activity of Tregs [124] and, consequently, in an increased overall immune response. It is conceivable that a combination of TAA modulation and induction of TNA by highly mutagenic anticancer drugs like temozolomide (see below) together with abrogation of the inhibitory function of Tregs by low dose cyclophosphamide could be able to provide full stimulation of the antitumor immune response required for triggering the abscopal effect.

### 3.1. Drug-Induced TAA Upregulation

Increased expression of TAA by pharmacological procedures has been the subject of several studies in the last 30 years, with the intent to suppress malignant cells utilizing selective immuno-cytotoxic approaches. A detailed analysis of TAA that can be considered for modulation by external agents has been reported previously [120]. Typical examples of drug-induced TAA modulation are discussed below.

#### 3.1.1. Interferons 

One of the first agents that was discovered to induce a considerable TAA up-regulation was interferon-gamma which was shown to substantially amplify CEA expression both in vitro [125,126,127] and in vivo [128]. IFN-α also increased CEA in vitro [127,129] and in carcinoma patients [130]. These agents are able to upregulate other TAAs, including tumor-associated CA125 antigens in ovarian cancer cells [131], glycoprotein-72 (TAG-72) in patients with colon [128,130] or breast cancer [132], and TAAs in glioma [133,134] or breast cancer cell lines [135]. Moreover, EpCAM, a TAA detectable in colon cancer cells, was also found to be upregulated by IFN-α and IFN-γ [136]. Tumor cell lines expressing Her-2/neu peptide presented by HLA-A*0102 showed upregulation of the Her-2(369)-A2 epitope after exposure to IFN-γ [137], and IFN-β was found to be capable of modulating TAA. In this report, upregulation of Melan-A/MART-1 gp-100 and MAGE-A1 in melanoma cell lines exposed to this cytokine was described [138]. Similar results were obtained in neuroblastoma cells concerning an increase in MAGE-A1, MAGE-A3, and NY-ESO-1 expression under the influence of IFN-γ and 5-aza-2’deozycytidine (decitabine) [139]. On the other hand, a report showing that IFN-γ downregulates the presentation of TAA in freshly prepared epidermal APC in mice [140] proposed that the effect of IFN-γ on TAA presentation may be tumor-specific and has to be checked for each tumor entity carefully.

#### 3.1.2. Epigenetic Drugs 

Gene transcription is finely tuned by the interaction of DNA with histone proteins that are organized in chromatin fibers. In the last few years, a number of drugs has been developed to regulate these interactions [141,142,143], including the inhibiting histone deacetylases (HDACs) that remove acetyl groups from the N-terminal lysine of nucleosome histones [144]. In principle, since histone deacetylation leads to gene silencing, it is reasonable to predict that HDAC inhibitors can upregulate TAA expression. This expression can also potentially be regulated through the HDCA-mediated modulation of both the mRNA elongation machinery [145] and the activity of transcription factors [146].

For example, valproic acid, a well-known HDAC inhibitor, is able to increase the expression of the CD20 antigen on B-cell lymphomas, thus increasing the complement-dependent cytotoxic effects of the anti-CD20 mAb rituximab [147]. It should be noted that IFN-β and valproic acid are also effective in sensitizing cancer cells to chemotherapeutics directly, which was attributed to the reactivation of silenced caspases [148]. Nevertheless, of particular interest are studies concerning the therapeutic potential of epigenetic drugs, including HDAC inhibitors, which are associated with ICIs in tumor immunotherapy approaches [149]. Actually, if tumor cells are scarcely immunogenic, no cytotoxic effector cells are produced by the tumor-bearing host. In this case, treatment with ICIs is irrelevant. However, if malignant cells acquire remarkable immunogenic properties under the influence of epigenetic drugs, it is reasonable to predict that ICIs will become highly efficient in eliciting anticancer effects.

DNA methyltransferase inhibitors 5-aza-cytidine and decitabine represent another group of epigenetic drugs that have been found to be able to support antitumor immunity by increasing TAA expression and presentation [139]. For example, in a model of murine pancreatic ductal adenocarcinoma, it was shown that azacytidine can substantially increase the TAA presentation to T cells, along with T-cell infiltration of the neoplasia and presumably amplification of target tumor immunogenicity [150]. The increase in CEA antigen expression in human colorectal tumor cell lines induced by 5-azacytidine renders malignant cells more susceptible to CAR-NK-mediated cytotoxicity targeting CEA in vitro and in SCID mice [151].

#### 3.1.3. Antitumor Agents

*Docetaxel*

Hodge et al. [152] showed that docetaxel is able to increase the expression of TAA (i.e., CEA, MUC-1, and PSA) in docetaxel-resistant cells, thus highlighting that TAA upregulation is not necessarily coincident with ICD (see the ICD section of this review). Therefore, the authors proposed calling this phenomenon “immunogenic modulation.”

*5 -Fluorouracil (5-FU)*

A number of in vitro experiments on human colorectal cancer cells demonstrated that this antimetabolite is able to substantially increase the expression of CEA epitopes on the tumor cell membrane in vitro [113,153,154,155,156,157,158,159,160,161,162]. This effect has been exploited to facilitate the detection of circulating colon cancer cells in patients [163,164]. In the case of melanoma cells, treatment in vitro with 5-FU sensitized cells to lysis induced by cytotoxic CD8+ T cells (CTL) recognizing the G209 antigen [165]. Moreover, as reported in the same study, treatment with dacarbazine produced similar effects. We should note that this finding needs confirmation in other cell systems since dacarbazine is a prodrug and not active in vitro without metabolic activation [166]. In cholangiocarcinoma cells, 5-FU, gemcitabine, and IFN-γ have been shown to upregulate MUC1 antigen expression and concomitantly MHC and PD-L1 [167], thus suggesting that an appropriate combination of chemotherapy and immunotherapy including ICIs might afford better results than chemotherapy alone. 

*Drugs Targeting Thymidylate Synthase*

In a number of preclinical and clinical investigations, it was shown that the thymidylate synthase (TS) could be considered a TAA since this enzyme is abnormally expressed in various neoplasia, mainly in colorectal cancer, which occurs especially under treatment with 5-FU [157,168,169,170,171,172,173,174].

*Platinum Compounds*

Colorectal tumor-associated antigen-1, mesothelin tumor-associated antigen, and telomerase catalytic subunit TERT were upregulated in oxaliplatin-resistant colorectal cancer cells collected from a large number of patients treated with the drug [175]. In vitro studies showed that oxaliplatin and other antineoplastic agents (e.g., 5-FU, mitomycin-C, and raltitrexed) upregulated membrane Lewis(y) antigen [136] in colorectal cancer cells. Whether this occurs during chemotherapy is yet to be demonstrated.

*Gemcitabine*

Investigations on the immune effects of gemcitabine revealed that this drug can upregulate death receptors on tumor cells, rendering them more susceptible to the cytotoxic effect of CD8+ T cells. Moreover, gemcitabine substantially upregulates NKG2D ligand in malignant cells, which become highly susceptible to killing by NK effector cells [176,177]. However, strictly speaking, this type of drug-mediated function cannot be classified as TAA upregulation, but rather as a drug-induced increase in tumor cell susceptibility to immuno-mediated killing.

### 3.2. Drug-Induced Xenogenization (DIX)

**Figure 2:**
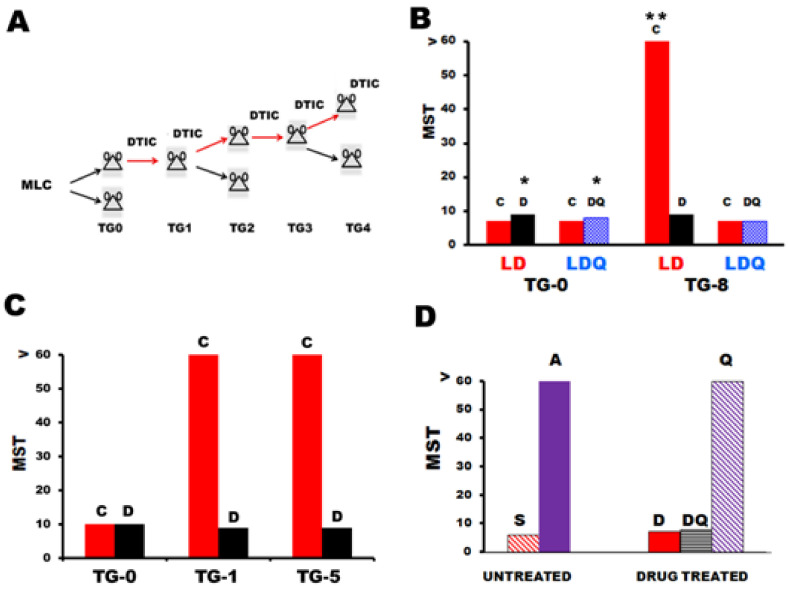
DTIC-induced xenogenization. Quinacrine interferes with DTIC-induced xenogenization, but not with the antitumor or immunosuppressive activity of this drug, or with the onset of resistance to the triazene compound. (**A**) Graphic protocol showing the treatment scheme utilized to expose in vivo mouse leukemia cells (MLC) to DTIC (100 mg/Kg ip, day 1–5, [178]) in the course of sequential transplant generations (TGs). At each TG recipient mice were left untreated or treated with DTIC for the subsequent TG. (**B**) Co-administration of DTIC + quinacrine abrogates the DIX effect afforded by DTIC alone [189]. LD line, L5178Y leukemia of DBA/2 origin, passaged (10^6^ cells ip) in CD2F1 mice treated with DTIC alone (D, black columns); LDQ line, same line passaged in mice treated with DTIC + quinacrine (Q, 20 mg/Kg ip, day 1–5 after challenge, blue letting columns). Red columns indicate survival times of untreated controls of LD or LDQ lines. Moreover, Q treatment alone was entirely inactive (data not shown). At TG8, the DIX effect was not detectable in the LDQ line but only in the LD line, which became strongly immunogenic (i.e., all 8 untreated controls lived more than 60 days, whereas the DTIC-treated mice died as a result of DTIC-induced immunosuppression [190]). Moreover, after several TGs, LDQ became entirely resistant to the antitumor effect of DTIC, thus confirming that Q did not prevent chemo-resistance to DTIC (data not shown). (**C**) Early DIX detectable in the LDQ line. CD2F1 mice were challenged with 10^6^ cells ip of the non-immunogenic LDQ line (collected from leukemic donors at TG9). Thereafter, the mice were treated with DTIC alone, and at the following TG1 the majority of untreated controls survived beyond the 60-day observation period. (**D**) Quinacrine does not prevent the immunosuppressive effects of DTIC. A total of 10^6^ cells of L5MF-22 lymphoma of B10.129(5M) (H-2^b^) origin were inoculated intravenously into untreated syngeneic recipients (S) or into H-2-incompatible allogeneic (A) CD2F1 (H-2^d^/H-2^d^) mice. All CD2F1 mice (blue column) rejected the tumor whereas all syngeneic recipients (red dashed column) died within 6–7 days. Allogeneic CD2F1 hosts treated with DTIC (50 mg/kg ip, daily, day 1 through 5 after tumor challenge) alone (D, red column), or associated with quinacrine (20 mg/Kg, day 1-5) DQ (grey columns), died of generalized lymphoma within 6–8 days after challenge, without any significant difference between the two groups. All allogeneic mice treated with quinacrine alone (Q, violet dash column) rejected L5MF-22 cells. **MST, median survival times.** Panel B: * *p* < 0.05 (Mann–Whitney “U” test) respective to untreated controls at TG 0. At TG8, ** *p*< 0.01. In all other panels the reported differences are highly significant (*p* < 0.01).

In 1970, it was shown for the first time by two of us that treatment of mice bearing fully histocompatible non-immunogenic L1210 leukemia cells (originating from DBA/2; H-2^d^/ H-2^d^) with dacarbazine was effective at inducing the appearance of novel and unexpectedly strong transplantation antigens [178]. Dacarbazine is a triazene compound (dimethyl-triazene-imidazolecarboxamide, DTIC) that needs metabolic activation in the liver to yield the ultimate reactive agent MTIC, generating carbenium ions that methylate the DNA [179]. The induction of TNA was observed following in vivo exposure of malignant cells to DTIC for a number of sequential drug treatments (Figure 2A and Figure 2B). The host’s immune response against DTIC-treated cells was found to be comparable to that elicited by MHC-incompatible malignant cells [19,180]. Actually, all intact (BALB/c xDBA2)F1 (CD2F1) mice (H-2^d^/ H-2^d^) were able to reject up to 10^7^ L1210/DTIC cells inoculated ip, whereas all CD2F1 mice succumbed to generalized leukemia when inoculated with a low number (about 10) of untreated L1210 cells within 14 days after challenge [19]. Conversely, all mice, which were immune depressed following total body irradiation or high-dose cyclophosphamide treatment before L1210/DTIC transplantation, developed leukemia and died. These data confirmed the immunological nature of the mechanism underlying L1210/DTIC rejection. Similar results were obtained with different mouse leukemia cell models treated in vivo [39,181,182,183,184,185] or in vitro [166,180] with metabolically activated DTIC or with other triazene compounds that were found to be even more active than DTIC, and in some cases not requiring metabolic activation in vitro [180]. This phenomenon was termed "chemical xenogenization" (CX) [186] and more recently renamed “drug-induced xenogenization” (DIX) [19], which underlines that strong immunogenicity was elicited by treatment with an antitumor agent of therapeutic interest. Actually, the DIX acronym was adopted to distinguish this type of xenogenization from that described later in 1976, elicited by the chemical methylating mutagen N-methyl-N’-nitro-N-nitrosoguanidine, which is not used therapeutically [187]. Other antitumor agents have been tested for DIX activity. However, in no case was the level of immunogenicity achieved comparable to that obtained following sequential treatment with triazene derivatives ([183,188] and unpublished data from our laboratory).

From the beginning of our observations on DTIC immuno-pharmacodynamics, we hypothesized that triazene-induced tumor immunogenicity could have been the result of drug-induced somatic mutations. This was supported by the finding that upon metabolic activation, DTIC is a highly mutagenic compound. It was further confirmed by subsequent studies on the mechanisms underlying triazene-dependent DIX. Epigenetic mechanisms relative to DNA methylation have been essentially ruled out since triazenes do not show hypomethylating activity, but rather a weak hypermethylating activity on DNA [191]. Moreover, it was found that CD2F1 mice rendered immunologically tolerant to weak virus-dependent antigens expressed by the moloney virus-induced LSTRA leukemia were able to reject DTIC-treated LSTRA cells [39]. This finding showed that the drug did not induce xenogenization of malignant cells through a mechanism based on the enhancement of virus-coded transplantation antigens [192]. Rather, it supported the hypothesis of neoantigen generation following in vivo or in vitro treatment of cancer cells with triazenes. 

Further experiments aimed at supporting this hypothesis were conducted by combining the administration of an antimutagenic compound such as quinacrine [193] with DTIC, as shown by Giampietri et al. [189] (see Figure 2). It was found that quinacrine suppressed entirely DTIC-induced xenogenization. Indeed, L5178Y leukemia cells (of DBA/2 origin) exposed in vivo to DTIC alone were rejected by intact non-DTIC-treated (i.e., non-immunodepressed) histocompatible CD2F1 recipients, whereas leukemic cells treated in vivo with DTIC together with quinacrine for 8 TGs were lethal for both intact and immunodepressed hosts ([189], Figure 2B). It must be stressed that quinacrine did not interfere with the antitumor activity of DTIC since leukemic mice treated with DTIC or with DTIC plus quinacrine at TG-0 survived modestly, but significantly longer than control animals (Figure 2B). Moreover, quinacrine did not antagonize the immunosuppressive effects of high doses of DTIC (Figure 2D) and did not prevent the induction of chemoresistance to DTIC when associated with the triazene compound [194]. Notably, murine leukemia cells that were highly resistant to DTIC and not immunogenic following serial passages in histocompatible mice treated with DTIC + quinacrine became highly immunogenic if exposed in vivo to only one cycle of DTIC without quinacrine (Figure 2C). All these findings indicate that quinacrine impairs selectively DTIC-dependent DIX and suggest that mutations causing DIX were different from those possibly provoking cellular resistance to the triazene compound. Although the molecular basis of this specific pharmacodynamic antagonism has not been clarified yet, the findings appear to disclose novel approaches to analyzing the mutation patterns involved in TNA generation.

The direct and conclusive demonstration that triazene-induced DIX is generated by somatic mutation was provided by studies performed by Grohmann et al. in 1995 [195]. They found that the immunogenicity of a clone of the L5178Y/DTIC cell line was due to somatic mutations in a specific region of a murine endogenous retrovirus. Moreover, they identified the MHC-presented non-self-peptide(s) responsible for the leukemia graft rejection.

#### 3.2.1. Mechanism of Action of Triazenes

**Figure 3:**
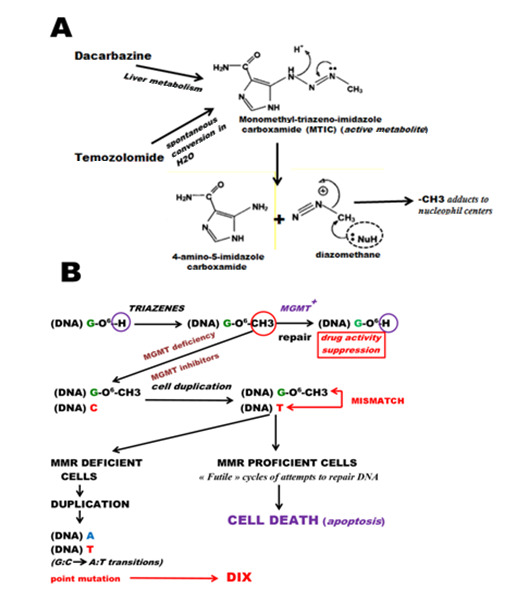
Biochemical basis of the DIX effect due to exposure to triazene compounds. (**A**) Dacarbazine (DTIC) is a prodrug that requires liver metabolism to be converted into the active MTIC metabolite. On the other hand, temozolomide, an often-used triazene compound, does not require metabolic activation since it is spontaneously converted into MTIC in aqueous solution. Thereafter, MTIC generates the final active molecule, diazomethane, a mono-methylating agent able to produce methyl adducts to nucleophil centers in the DNA. (**B**) The most significant methyl adducts to DNA produced by triazenes relative to their pharmacodynamic properties include O^6^MeG, N7-methylguanine, and N3-methyladenine [209]. At therapeutic dose levels, particularly involved in the cytotoxic and DIX effects of triazenes is O^6^MeG. If this adduct is repaired by MGMT, triazenes are essentially inactive [19,210,211]. If the activity of MGMT is spontaneously low or downregulated by drugs (e.g., lomeguatrib [212]) O^6^MeG mispairs with thymine. If MMR is functionally active, the complex tries to repair the mismatch by replacing the new DNA strand without success. It is presumed that several “futile cycles” of DNA repair lead to cell death through apoptosis. However, a few resistant clones that are inefficient for MMR or that do not trigger apoptosis continue to proliferate and display point mutations (GC->AT), giving rise to the appearance of strong tumor neoantigens (i.e., the DIX effect).

Triazene derivatives (e.g., DTIC and TMZ) and hydrazine derivates (e.g., procarbazine) are highly mutagenic compounds. Different from DTIC and procarbazine, TMZ does not need metabolic activation. The main biochemical mechanisms underlying the mutagenic activity of triazenes [196] leading to DIX is illustrated in Figure 3. Upon metabolic activation of dacarbazine, which occurs predominantly in the liver, the reactive metabolite MTIC is formed, which is released into the blood stream and enters tumor cells by diffusion. In the cell, the metabolite decays spontaneously, yielding carbenium ions that methylate the tumor DNA at different sites of the purine and pyrimidine bases. One of the DNA methylation products is O^6^-methylguanine (O^6^MeG). Although formed in minor amounts (maximally 8% of total alkylations), it is responsible for most of the point mutations induced by these agents, since O^6^MeG has highly mispairing properties, pairing with thymine during replication [197]. This is followed by GC-to-AT transition mutations. For this process, replication is required, which implicates that proliferating tumor cells exhibit a high mutation rate. There is another important condition that is required for successful mutagenicity (Figure 3B), namely, the absence or strongly reduced activity of the DNA repair protein O^6^-methylguanine-DNA methyltransferase (MGMT). This protein removes the methyl group from the O^6^-position of guanine through transfer to an internal cysteine and thus restores guanine in the DNA. Therefore, it prevents point mutations induced by DNA-methylating drugs. It is interesting that MGMT is highly regulated and silenced in many tumors [198,199]. Thus, in malignant glioma (grades III and IV), MGMT is completely lacking in about 20% of the tumors and epigenetically downregulated in about 40% of neoplasias, resulting in a significant decrease in MGMT repair activity [200,201,202]. A process that counteracts mutagenicity through O^6^MeG is mismatch repair (MMR), as O^6^MeG:T mismatches are recognized by MMR proteins (MSH2/MSH6) [203] and erroneously processed in a futile repair cycle, yielding DNA double-strand breaks that trigger cell death [204] and senescence [205]. Through this process, cells harboring premutagenic changes can be eliminated. However, all experimental systems in which mutations through O^6^MeG were measured showed that MMR does not eliminate all premutagenized cells, and mutations increase linearly with dose and O^6^MeG level [206]. It is also conceivable that in cancer cells apoptotic processes are blocked or defective, e.g., through upregulation of antiapoptotic factors such as survivin, BCL-2, or lack of caspases [207,208]. In this case, mutated cells will survive and propagate, and finally express mutated proteins followed by TNA presentation.

Based on these considerations, it is obvious that MGMT downregulation appears to be mandatory for DIX achievement with triazenes. It must be underlined that the majority of preclinical studies on DIX have been conducted on mouse leukemia/lymphoma cells that express low levels of MGMT [213,214]. Actually, there are a number of drugs able to inhibit or downregulate MGMT activity [210,212,215,216,217]. Moreover, triazenes themselves inactivate the suicide enzyme MGMT due to the repair reaction, which leads to MGMT depletion after repeated treatment cycles. There are also other anticancer drugs that may impact MGMT expression, such as cisplatin [218], bortezomib [219,220,221], and PARP inhibitors [222], which were reported to downregulate MGMT activity. However, the difficulty with these genotoxic agents lies in the additive toxic effects produced when combined with triazene compounds such as DTIC and TMZ. However, highly specific, potent, and non-toxic MGMT inhibitors have been developed over the past 20 years. The classical inhibitor utilized in many in vitro and in vivo preclinical studies is O^6^-benzylguanine (O^6^BG) [223,224]. This compound is a pseudo-substrate that binds MGMT, forming S-benzylcysteine at the acceptor site of MGMT and thus inactivating the repair protein. Thereafter, inactivated MGMT undergoes ubiquitination and degradation so that its activity can be restored only after de novo synthesis of the protein. Another MGMT inhibitor, 6-(4-bromo-2-thienyl) methoxylpurin-2-amine (lomeguatrib) [210,215,224,225,226], exhibits a similar mechanism of action. It has a stronger affinity to MGMT and is therefore about 10-fold more active than O^6^BG [210]. Importantly, both inhibitors are essentially non-toxic and are well tolerated, as shown in cultivated cells, animal systems, and in clinical trials [227]. In detail, these clinical studies showed that lomeguatrib exhibits minimal toxicity and an extremely high suppressing effect on MGMT in leukemia [209,228,229], melanoma [230,231,232], and colorectal cancer [233]. However, systemic administration leads to MGMT inactivation not only in the tumor, but also in the healthy tissue throughout the body. Therefore, MGMT inhibitors not only amplify the antitumor activity of TMZ, but also increase its toxic side effects, notably hematotoxicity [234]. Therefore, coadministration of methylating anticancer drugs and MGMT inhibitors did not result in an improved therapeutic index compared to TMZ alone. Although similar studies have not been performed with dacarbazine, similar outcomes are anticipated. Therefore, MGMT inhibitors do not appear to be manageable in high-dose cancer therapy with alkylating agents, unless they are locally administered, as shown in a therapeutic approach for glioblastoma [235]. In addition, strategies are available for hematoprotection through the transfer of a mutated form of MGMT, which is resistant to O^6^BG, in hematopoietic stem cells prior to high-dose TMZ therapy [236]. Other targeting approaches have been discussed that are still in the preclinical stage [227]. Nevertheless, MGMT inhibitors appear to be mandatory in MGMT-expressing tumors for DIX-dependent cancer therapeutic approaches in which TMZ or dacarbazine are applied to induce cancer cell immunogenicity. Actually, persistent MGMT inactivation has been obtained with protracted low-dose administration of TMZ [237]. As continuous low-dose TMZ has been shown to induce low yet detectable levels of mutations (phase II trial with recurrent malignant glioma patients [238]), a metronomic schedule involving low-dose TMZ is likely to represent an optimal option for accumulating immunogenic mutations notably in tumors in which MGMT is epigenetically silenced.

In conclusion, since TNAs rest on the expression of new proteins resulting from mutations, it is reasonable to postulate that genotoxicants with high mutagenic activity and low cell-killing ability are efficient TNA inducers. As previously stated, an example is N-ethyl-N-nitrosourea (ENU), which is highly mutagenic and only slightly cytotoxic. Although not employed in cancer therapy, it might be considered a powerful TNA inducer. Moreover, the DNA methylating agent streptozotocin, a glucose conjugate with N-methyl-N-nitrosourea, TMZ, dacarbazine, and procarbazine, is also highly mutagenic, although clearly more toxic, at least in MGMT-deficient cells [239,240]. It is utilized in cancer therapy and therefore can be considered to be applicable for TNA induction. We should note that ENU and MNU are highly carcinogenic agents, which may limit their application in cancer therapy.

#### 3.2.2. DIX and Ionizing Radiation

**Figure 4:**
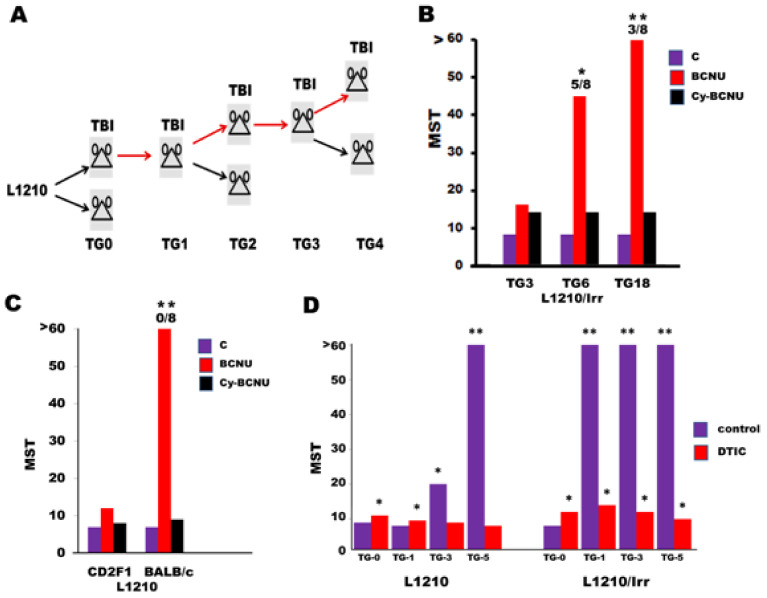
Radiation-induced increased immunogenicity and DIX in untreated or radioresistant mouse leukemia cells. (**A**) Protocol used to obtain radiation-resistant L1210 leukemia sublines collected from mice subjected to total-body irradiation (TBI, 4 Gy) on day +1 after inoculation of 10^6^ L1210 cells ip [242]. (**B**) Increased immunogenicity of L1210/Irr subline; CD2F1 mice bearing non-irradiated L1210 cells (TG0) showed an MST of 8.5 days, whereas irradiated mice showed an MST of 11.5 days (P < 0.05 Mann–Whitney U test, data not shown). Thereafter, no significant difference was detected between irradiated and non-irradiated recipients for up to 18 TGs (data not shown), indicating that the subline L1210/Irr became radioresistant but not noticeably immunogenic. In order to detect weak transplantation antigens, we applied a treatment protocol that takes advantage of synergistic effects between chemotherapy and marginal graft responses [243]. Intact mice bearing L1210/Irr cells obtained from TG6 or TG18 and treated with BCNU (red columns) lived significantly longer than immunodepressed recipients (black columns), indicating a weak host graft response against L1210/Irr line. (**C**) Immuno-chemotherapy synergism [243,244]. A total of 10^5^ cells of L1210 leukemia were inoculated ip into histocompatible CD2F1 mice or into BALB/c hosts incompatible with minor histocompatibility loci. No difference was found in the MST of histocompatible or allogeneic hosts (violet columns), which all died of leukemia. However, after treatment with BCNU (3.9 mg/Kg ip on day +3, red columns) histocompatible mice lived slightly longer than untreated controls, whereas all allogeneic BALB/c recipients (i.e., 8–12 mice) survived beyond the 60-day observation period. On the other hand, the MSTs of mice immunodepressed by Cy (150 mg/Kg, 8 h before tumor challenge) and treated with BCNU (black columns) were comparable in histocompatible and allogeneic mice. The number of dead mice over the total is indicated at the top of the columns. When not indicated, all animals died from leukemia (panels B and C). * *p* < 0.05, ** *p* < 0.01 (Mann–Whitney “U” test) considering BCNU vs. Cy-BCNU groups. (**D**) Comparative DTIC-induced xenogenization of the original L1210 leukemia and in vivo-irradiated L1210/Irr subline collected after 16 transplant generations of TBI. As obtained in a number of similar experiments, at TG5 and onward, L1210 cells exposed to DTIC (100 mg/Kg/day ip for 5 days) became strongly immunogenic and all non-treated controls (violet columns) rejected malignant cells and survived beyond the 60-day observation period. Surprisingly, in vivo-irradiated and radioresistant L1210/Irr cells underwent an early xenogenization process since leukemia cells acquired strong immunogenicity, responsible for total graft rejection, after only one cycle of DTIC treatment. Moreover, the same degree of immunogenicity was maintained for a number of additional TGs of DTIC treatment. The number of dead mice over the total is indicated at the top of the columns. *p < 0.05, ** *p* < 0.01 (Mann–Whitney “U” test) considering control vs. DTIC-treated groups.

Very limited information is currently available on the possible interaction between drugs endowed with DIX activity and RT. The main preclinical and clinical literature on this subject concerns the combined effects of TMZ and RT in brain tumors (recently reviewed [241]). Preclinical studies performed in one of our laboratories with L1210 cells showed an interesting phenomenon [242] for which the underlying molecular basis is still unknown. The results of pooled published experiments are shown in Figure 4. L1210 cells of DBA/2 origin inoculated into CD2F1 mice were exposed in vivo to γ-rays (total body irradiation, 4 Gy) for a number of sequential transplant generations, thus obtaining the L1210/Irr line (Figure 4A). At the end of the first treatment, irradiated mice lived significantly longer than non-irradiated recipients. However, after a few TGs, L1210/Irr cells became completely resistant to in vivo irradiation (data not shown). From TG6 onward, L1210/Irr cells showed a modest degree of immunogenicity revealed by the protocol based on immuno-chemotherapy synergism illustrated in Figure 4C. Leukemia cells collected from irradiated donors at TG6 or TG18 and inoculated into intact or immunodepressed (cyclophosphamide, 150 mg/Kg administered 8 h before tumor challenge) mice killed all hosts with similar MST (Figure 4B). However, if recipient mice were inoculated with BCNU ((3.9 mg/Kg ip, on day +3 after challenge), intact hosts lived significantly longer that immunodepressed recipients, whereas no difference in MST was found in intact or immunodepressed mice bearing untreated L1210 cells. Similarly, as shown in Figure 4C, L1210 leukemia inoculated into fully histocompatible CD2F1 mice or into BALB/c mice incompatible with minor histocompatibility loci showed the same MST in spite of a modest transplantation immunity of allogeneic hosts. If recipient mice were treated with BCNU, all histocompatible mice showed a limited increase in MST over that of untreated controls. However, if allogeneic BALB/c mice were treated with BCNU, all recipient animals were long-term survivors, whereas immunosuppressed hosts died with MST similar to that of histocompatible CD2F1 recipients. These results, confirmed by a number of experiments using various host/tumor systems, point out that this immuno-chemotherapy model is adequate to reveal the existence of limited host-anti-leukemia immune response. 

Much more noteworthy are data shown in Figure 4D. Radiation-resistant L1210 leukemia cells obtained from donors bearing the L1210/Irr line at 16 TG of irradiation, inoculated into CD2F1 mice and treated with DTIC, acquired strong immunogenicity only after a single cycle of DTIC treatment instead of the five cycles usually required with the parental L1210 cells. Therefore, it is conceivable that irradiation allowed the early appearance of a high yield of drug-treated immunogenic clones. We hypothesize that this effect is of therapeutic value in the course of the “dynamic dormancy” [245] of micro-metastases that could persist after tumor RT and generate a disease relapse. Cancer “dynamic dormancy” refers to the presence of quiescent malignant cells, surviving initial therapeutic treatment and seeded in different organs. Following appropriate stimulatory signals, these cells regain their ability to proliferate, ending in clinically detectable metastasis. In case of RT-treated dormant cells, it can be speculated that they could be particularly susceptible to DIX, as suggested by data illustrated in Figure 4, providing a rational basis for novel therapeutic approaches.

## 4. Immunogenic Cell Death

Immunogenic cell death (ICD) defines the mechanism by which physically or chemically induced cell death provokes a longstanding T-cell-mediated adaptive immune response. ICD can be considered a mainstay of the abscopal effect triggered by local treatment with ionizing radiation. The concept of ICD that emerged at the beginning of this century [246] was recently reviewed as a general phenomenon [247] and in relationship to RT [248] (for consensus guidelines see Ref. [249]). It should be noted that tumor cell death induced by therapeutic procedures is not necessarily immunogenic since the TME of dying malignant cells can be markedly immunodepressive [250]. Successful ICD is conditioned by the presence of TNA or by adequately high levels of non-specific TAA that can be preferentially targeted by an adaptive immune system [251]. For this reason, DIX based on TNA induction and ICD plays two different roles in tumor immunology. Indeed, DIX can take place in cells entirely resistant to the cytotoxic effects of the chemical agent triggering the xenogenization process [194]. On the other hand, ICD represents an important mechanism involved in the host’s antitumor immune responses elicited by cell death. An update of the principal factors regulating a functional definition of ICD along with procedures for its analysis has been provided by Fucikova et al. [252].

As described in Figure 4, cytotoxic lesions produced either by RT or chemotherapy orchestrate a series of pre-apoptotic or post-apoptotic intracellular and secretory signals collectively termed as damage-associated molecular patterns (DAMP) [253]. One of the main events that can be detected in the early stages of ICD is the translocation of calreticulin from the cytoplasm to the tumor cell membrane [254,255], which provides macrophage stimulation via the “eat me” signal that is antagonized by CD47 expressed on the tumor cell membrane [256]. Additional events have been considered, such as DC maturation [257], which in turn causes antigen-specific CD8^+^ T-cell proliferation through the release of ATP, annexin A1 (ANXA1), and type 1 interferon [253]. Moreover, of primary importance for ICD is the increased expression of MHC [159] accompanied by amplified levels of non-specific TAA or highly specific TNA (see Section 3). A further critical component of DAMP is represented by high-mobility-group box 1 (HMGB1) protein [253,258] that is released by apoptotic cells and is involved in different physiological and pathological processes, including those mediated by NK and CD8+ T-cell-mediated cytotoxicity [259] or the induction of malignant cell proliferation [260]. 

Several investigations indicated that ICD synergizes with ICI to additionally increase tumor-specific T-cell functionality, suggesting that ICD is able to transform tumor cells into an endogenous vaccine, improving the clinical outcome of ICI therapy [261,262]. However, despite the large amount of data obtained on the mechanism underlying ICD, there is still inadequate information available about the category of antigens that become immunogenic in response to ICD [263].

Traditional chemotherapy still represents an invaluable option in cancer treatment, and can also play a potential critical role in improving tumor-specific T-cell functionality through the generation of ICD [264]. The old concept that antineoplastic chemotherapy is only detrimental to the anti-tumor immune response has been challenged by recent studies showing that it can improve both innate and adaptive responses with different mechanisms [262]. The immunogenic effects of chemotherapeutic drugs have been underestimated for a long time, since experimental models have mainly employed immune-deficient animals without dedicating the effort they deserve to the effects of anticancer agents on different features of the immune response. However, the mechanisms underlying chemotherapy-induced cancer regression cannot be ascribed simply to the cytotoxic and cytostatic effect of anticancer drugs, but have to also consider the effects mediated by tumor-specific T-cell responses [265,266]. 

Remarkably, alongside the DIX effect, chemotherapy is also able to intensify tumor cell immunogenicity by stimulating the expression of MHC-I molecules [267]. Moreover, cytotoxic agents could provide additional anti-cancer mechanisms, including the activation of NK cells by stimulating the specific NKG2D ligand and the induction of DC differentiation as well as the improvement of T-cell functionality. All these events are also related to the eradication of myeloid-derived suppressor cells (MDSC) and Tregs along with the induction of a "cytokine storm" [268,269,270,271,272,273], as reported in protocols combining anti-cancer vaccination and chemotherapy [274]. It is important to note that the major antigen-presenting cells, DCs and macrophages, are quite resistant to anticancer drugs such as TMZ- [275,276] and ROS-generating treatments, including ionizing radiation, compared to T cells [82,277]. Therefore, it is conceivable that they remain functionally active during therapy, stimulating the anti-tumor immune response even in a high-dose therapeutic setting.

Unfortunately, several drugs, including cisplatin, DTIC, 5-FU, gemcitabine, irinotecan, oxaliplatin, paclitaxel, and others, show the ability to upregulate PD-L1 expression on cancer cells through the generation of danger signals [278]. In contrast to this, capecitabine was found to inhibit the expression of CTLA-4 in colorectal cancer cells [279], and the anti-tumor activity of orlistat, an FASN inhibitor, has been associated with the reduction of PD-L1 expression [280]. These data indicate that chemotherapeutics have the ability to modulate the antitumor immune response by impacting the expression of important receptors regulating cytotoxic T-cell activity.

The effects of chemotherapeutic drugs can be classified according to their ability to cause or support ICD. For example, alkylating drugs can modify T-cell activation. In particular, cyclophosphamide, which is immunosuppressive in high doses, impairs regulatory Tregs when delivered in terms of metronomic schedules [269,281]. Remarkably, when provided as metronomic repeats every 6 days, cyclophosphamide impairs tumor growth and activates robust anti-tumor immune responses in both SCID (adaptive immune-deficient) and fully immune-competent C57BL/6 mice [282]. 

Similar responses have been reported for TMZ. Although treatment with a standard dose of TMZ plus radiation in GBM patients was associated with a strong T-cell decline, low-dose metronomic administration of the drug was associated with fewer circulating Tregs and a reduced extent of CD8+ T-cell exhaustion [283]. Therefore, a metronomic schedule involving cyclophosphamide or TMZ treatment in association with RT can be considered a strategy for generating immunogenic mutations without compromising the anti-tumor immune response, thus supporting ICD.

Since distinctive settings have been shown to be differently responsive to drug-mediated ICD, it is conceivable that insensitive neoplasias do not possess the intrinsic features required for ICD induction [284]. An example is given by platinum compounds. Although oxaliplatin strongly induces ICD [284,285], cisplatin is devoid of intrinsic ICD activity. However, the drug induces ICD when combined with N-(2-hydroxypropyl) methacrylamide (HPMA) copolymer (P-Cis) and digoxin [286]. Another example is 5-FU, which, besides being a TAA upregulator, decreases the frequency of MDSCs [287] and induces T-cell infiltration and functionality in colorectal cancer patients along with a better clinical outcome [288]. Remarkably, both anthracyclines and bleomycin are able to improve the host immune function through the induction of ER stress, leading to positive patient responses [289,290]. 

Radiotherapy delivered at clinically significant dosages is able to stimulate signaling pathways leading to ICD [291], although the immunostimulating effects are often counteracted by the detrimental tumor-associated milieu [292,293], a condition that can potentially be counteracted by an IC blockade [294,295]. However, as noted above, the RT dosage and the specific schedule of treatment are both critical factors in order to obtain proper tumor-derived antigens able to elicit an adequate T-cell-mediated immune response through ICD and the abscopal effect. Nevertheless, the highest level of immunostimulation was obtained using a fractionated schedule, and this was further potentiated by ICI co-administration [294,295]. Overall, RT is potentially able to switch the mechanisms underlying the effect of traditional chemotherapeutics towards ideal ICD inducers [296]. 

We should note that autophagy, known to interact in a complex manner with ICD [297], can be upregulated in malignant cells (e.g., in prostate cancer under the influence of AMBRA1 [298]), which may result in the inhibition of tumor cell death. This may attenuate abscopal tumor-eliminating effects. Therefore, it is conceivable that in the case of autophagy upregulation [299] autophagy inhibitors might contribute to enhancing the abscopal effect determined by RT and supported by chemically induced ICD.

Only a limited number of actual ICD activators has been used efficiently in clinical practice [300,301]. These agents can potentially play a critical role in triggering the anti-cancer immune responses that can be enforced by immunotherapies in the setting of combined therapeutic strategies [302,303]. According to these observations, several ICD stimulators are currently under investigation in a situation of off-label employment for cancer treatment in combination with ICI [304,305]. The purpose of upcoming investigations should be aimed at evaluating how traditional chemotherapies and new anticancer drugs collaborate with RT in converting the TME from an immunosuppressive to a highly responsive pattern.

## 5. Conclusions

**Figure 5:**
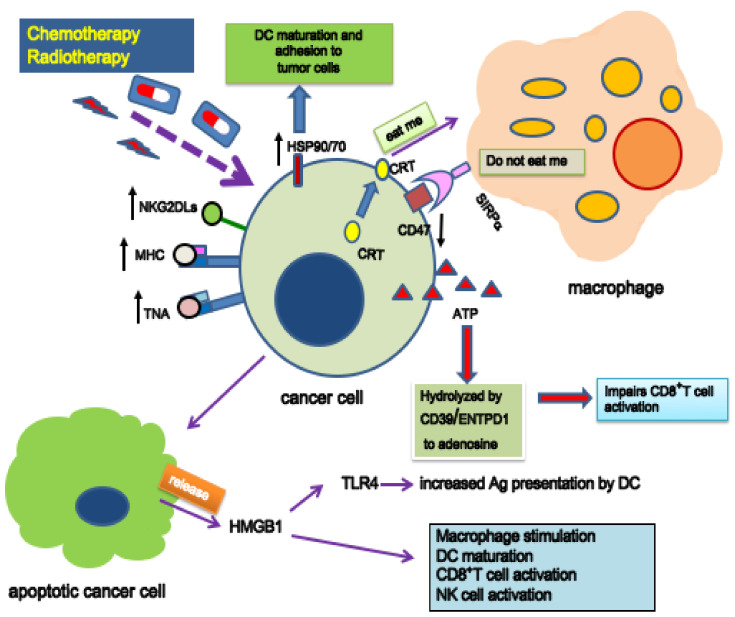
Immunogenic cell death induced by chemotherapy or radiotherapy; possible biochemical patterns. Translocation of calreticulin (CRT) [255] from cytoplasm to tumor cell membrane, facilitated by annexin [306]. Surface CRT is able to induce an “eat me” signal to phagocytes [307], increasing their activity along with DC maturation and antigen-presenting function [308]. The calreticulin effect is antagonized by the interaction of CD47 present on the tumor cell membrane with SIRP-alpha α located on macrophages and DC [309]), generating a “do not eat me” message in target phagocytes [310]. Increased expression of HSP90 and HSP70 by ICD inducers [247]. This is followed by DC maturation and adhesion to tumor cells with consequent enforcement of the host’s cell-mediated immune responses. The augmented expression of MHC and MHC-presented TNA is triggered by ICD inducers [247] and, indirectly, by interferons that are induced by the same agents. Moreover, agent-induced upregulation of NKG2DLs on the tumor cell membrane increases antitumor NK cell activity following interaction with NKG2D receptors present on NK cells [311,312]. Reduced expression of CD47 along with CD46 and CD31 are normally involved in “do not eat me” signaling [313]. The release of ATP in the tumor environment during ICD activates P2RX7 purinoreceptors present on DC. This is followed by the activation of CD8+T cells. This functional response is inhibited by CD39/ENTPD1 that hydrolyzes ATP [83].

Experiments by us and others revealed that (a) certain anticancer drugs, particularly triazenes, induce the appearance of TNA (i.e., DIX); (b) other chemical agents and certain cytokines amplify the expression of TAA and TNA; (c) ionizing radiation provokes the “abscopal effect” if delivered under particular conditions, generating a sort of “in situ vaccine” [20,301]; and (d) ionizing radiation and cancer chemotherapy can afford ICD, which contributes efficiently to the host immune response against malignant cells (see Figure 5). The described features are anticipated to substantially improve the antitumor immune response elicited through ICI. 

Major concerns that require further investigation result from the extremely complex interaction among all the players of this intricate network. Keeping in mind that DIX is the consequence of drug-induced somatic mutations, no data are currently available that indicate a way of amplifying DIX, with the exception of an appropriate use of ionizing radiation. To our knowledge, no studies have been performed to establish whether DIX can be amplified by epigenetic drugs. Actually, we know that pre-irradiation of target leukemia cells consistently reduces the number of treatment cycles with triazenes required to generate highly immunogenic blasts in the murine model illustrated in Figure 4. However, radiation exposure may increase MGMT levels in cancer cells, as demonstrated for rat hepatoma [314]. It is still unclear whether this occurs in human tumors as well. Although studies on cultivated human glioblastoma cells proved negative [315], it cannot be excluded that this occurs in vivo. Given the case that MGMT is subject to upregulation in human tumors following radiotherapy, a reduction in the antitumor and xenogenizing effects of triazene compounds can be expected [19,211]. Is should be noted that this negative outcome was not observed in the murine model since mouse L1210 leukemia does not express MGMT [211]. Moreover, RT upregulates PD-L1 expression in malignant cells [110] with consequent immune escape of target cells [316]. In this case, the effect of radiation can be antagonized by treatment with selective ICIs such as anti-PD-1 or anti-PD-L1 mAbs [316].

It is well known that an increase of mutation frequency results in enhanced intra-tumor heterogeneity (ITH) [317], which is supposed to have negative consequences on antitumor immune responses [119]. In a glioblastoma model, it was shown that ITH can be considerably reduced by exposure of malignant cells to ionizing radiation that downsized the clonal diversity by selecting radiation-resistant clones [318]. Therefore, it is reasonable to predict that pharmacological approaches may also reduce ITH in order to amplify the therapeutic value attainable with DIX-based antineoplastic immuno-radiotherapy treatments.

Natural immunity must be also considered as a potential target of radio-immuno-chemotherapy of cancer. Only preliminary results have been obtained regarding the relationship between DIX and natural antitumor host resistance. In our laboratory we have shown that *Hh-*type natural resistance detectable in lethally irradiated mice (according to Cudkowicz [319]) could contribute to in vivo resistance of mice against histocompatible [320] or allogeneic [183] malignant cells treated with antitumor agents, including dacarbazine.

In conclusion, we hypothesize that a therapeutically relevant abscopal effect may result from a proper combination of drug treatment, patient-adapted RT, and immunotherapy. Although there is still a long way to go to clinical application, the alliance of DIX and RT-induced abscopal effect may provide a significant contribution to cancer treatment.

## Supplementary Materials

The following are available online at https://www.mdpi.com/article/10.3390/ijms221910672/s1.

## Abbreviations

A2AR adenosine A2A receptor; ANXA1 annexin a1; APC antigen-presenting cells; BATF basic leucine zipper ATF-like transcription factor; BCNU 1,3 bis-(2-chloroethyl)-1-nitrosourea, carmustine; CEA carcinoembryonic antigen; cGAMP cyclic dinucleotide 2’ 3’-cyclic GMP-AMP complex; cGAS cyclic GMP-AMP synthase; CRC colorectal cancer; CTLA-4 cytotoxic T-lymphocyte antigen 4; CX chemical xenogenization; Cy cyclophosphamide; DAMP damage-associated molecular patterns; DC dendritic cells; DsDNA double-stranded DNA; DsRNA double-stranded RNA; DIX drug-induced xenogenization; DTIC dimethyl-triazene-imidazolecarboxamide, dacarbazine; ENU N-ethyl-N-nitrosourea; ER endoplasmic reticulum; FASN fatty acid synthase; GBM glioblastoma multiforme; HDAC histone deacetylase; HIF-1hypoxia-inducible factor-1α, subunit alpha; HMGB1 high-mobility-group box 1; ICD immunogenic cell death; ICI/ICIs immune checkpoint inhibitor/ors; IKK inhibitor of nuclear factor-κB (IκB) kinase (IKK) complex; IRF3 interferon regulatory factor 3; IRF7 interferon regulatory factor 7; IT immunotherapy; ITH intratumor heterogeneity; LMG lomeguatrib; MAVS mitochondrial antiviral-signaling protein; MDA5 RIG-1-like receptor melanoma differentiation-associated protein 5; MDSC myeloid-derived suppressor cells; MGMT O^6^-methylguanine-DNA methyltransferase; MHC-I major histocompatibility complex (MHC)-1; MMR mismatch repair; MTIC 5-[3-methyl-triazen-1-yl]-imidazole-4-carboxamide; O^6^BG O^6^-benzylguanine; O^6^MeG O^6-^methylguanine; OS overall survival; PD-L1 programmed death-ligand 1; Q quinacrine; RIG-1 retinoic acid-inducible gene-1; 5-FU 5-fluorouracil; RT radiotherapy; SABR stereotactic ablative radiotherapy; STING stimulator of interferon genes; TAA tumor-associated antigens; TAG-72 glicoprotein-72; TAM tumor-associated macrophages; TBK1 tank-binding kinase 1; TERT telomerase reverse transcriptase; TG transplant generations; TMB tumor mutation burden; TME tumor microenvironment; TNF tumor necrosis factor; TMZ temozolomide; TNA tumor neoantigens(s); TRAF TNF receptor-associated factor; TS thymidylate synthase.
