# Supplementary material for: Abscopal Effect and Drug-Induced Xenogenization: A Strategic Alliance in Cancer Treatment?
Source: Int J Mol Sci. 2021 Oct 1;22(19):10672. doi: 10.3390/ijms221910672 (PMC8509363; doi:10.3390/ijms221910672)
Supplement: Supplementary file 1 [file ijms-22-10672-s001.zip › ijms-1350957-Supplementary Materials.pdf]

Table S1. Studies showing the abscopal effect in different clinical settings.

AUTHORS	STUDY TYPE	TYPE OF CANCER (n. pts)	STAGE	LOCATION OF METASTASES	RADIOTHERAPY	SITE TREATED	CONCOMITANT THERAPY	ABSCOPAL RESPONSE
Ye et al. [1] 2021	Phase I clinical trial	Lung cancer (31)	Progressive advanced		HFRT	Selected lesions	Nivolumab	HFRT increased response to nivolumab; OOR: 39.13%; CR rates: 13.04%
Bassanelli et al. [2] 2021	Observational, retrospective study	NSCLC (95)	Advanced		RT		Nivolumab	RT during Nivolumab: better outcomes; Median OS: 11.9 months
Maity et al. [3] 2021	Phase I trial	Melanoma (22)	IV		RT		Ipilimumab	5/22: partial response; 3/22: stable disease
Mattes et al. [4] 2021	Trial PCT	NSCLC (35)	Metastatic		SBRT	A single extracranial target lesion	Checkpoint inhibitor alone (19) or in combination with Carboplatin/pemetrexed (16)	15 pts: grade 1 to 2 toxicities (pneumonitis and fatigue); the best ORR1: 53%
Li et al. [5] 2021	Retrospective study	Solid tumors (32)	Metastatic		Hypofractionated RT		PD-1 inhibitor	RT+immunotherapy: no AE
Kazandjian et al. [6] 2021	Pilot study	Relapsed/refractory multiple myeloma			Hypofractionated RT	Focal lesion	Avelumab	RT+ CI: no apparent anti-tumor response
Margulis et al. [7] 2021	Phase II Trial	RCC (6)	3/6 Metastatic	Inferior vena cava (IVC) tumor thrombus (TT)	SABR	IVC-TT		1/3 metastatic pts: CR; 2/3: PR
Segal et al. [8] 2021	Phase II study	CRC (24)	Metastatic		EBRT		Durvalumab+Tremelimumab	Rare cases of AE
Theelen et al. [9] 2021	2 clinical trials (ph 2: PEMBRO-RT; ph 1/2: MDACC)	NSCLC (148)	Metastatic		EBRT	Lung metastases, lung primary disease and intrathoracic lymph nodes	Pembrolizumab (76 only pembro; 72 pembro+radiotherapy)	ARR: 19.7% with pembro.; 41·7% with pembro.+RT. ACR: 43.4% with pembro.; 65.3% with pembro.+RT

Singh et al. [10] 2020	Data evaluation	NSCLC	Metastatic	Brain	SRS		35% of patients received immunotherapy; 46% chemotherapy; 18% EGFR/ALK targeted therapy	Immunotherapy+SRS: improved PFS compared to other therapies
Lieverse et al. [11] 2020	Phase II clinical trial	NSCLC (126)	IV		SABR		L19-IL2	Their Phase I study: response in 2/6 pts; results of this Phase II study: expected in 2023
Funck-Brentano et al. [12] 2020	Retrospective analysis	Melanoma (26)	Advanced		Hypofractioned RT (n 23)-Palliative RT (n 2)-Brain radiosurgery (n 1)		Pembrolizumab or Nivolumab	AE in 35% of pts
Yano M et al. [13] 2020	Case report	Endometrial Carcinosarcoma (1)	Metastatic	Bone,lung and mediastinal LN	EBRT	Pelvis	Pembrolizumab	CR lasted for 2 months
Watanabe T et al. [14] 2020	Case report	Melanoma (2)	Metastatic	Case1: liver, muscle, groin<br><br>Case 2: liver, lung, abdominal LN	SBRT<br><br>SBRT	Liver<br><br>Liver, LN	Nivolumab<br><br>Pembrolizumab	CR<br><br>CR lasted for 2,5 years
Ellerin BE et al. [15] 2020	Case report	Parotid gland cancer (1)	cT3 cN2b M1 (IVC)	Cervical LN, lung, hilar, subcarinal and paratracheal LN	VMAT	Parotid mass	No	Reduction: lung lesions; regression: LN lesions lasted for 1 year

Choi JS et al. [16] 2019	Case report	Case 1: HNSCC<br><br>Case 2: SCC of larynx	Metastatic<br><br>Metastatic	Parotid, neck, mediastinum, hilum LN, liver, lung, stomach, adrenal, peritoneum<br><br>Neck dermal, neck and axillary LN	SBRT<br><br>SBRT	Submandibolar and neck LN<br><br>Axilla	Atezolimumab<br><br>Panitumumab+pembrolizumab	CR lasted for 13 months<br><br>CR lasted for 20 months
Garelli E et al. [17] 2019	Case report	Case 1: LCNEC<br><br>Case 2: Lung adenocarcinoma<br><br>Case 3: Lung adenocarcinoma	cT4N0M1b<br><br>cT2N3M1c<br><br>cT3N2M1a	Adrenal gland, spine<br><br>Mediastinal LN, brain, ocular<br><br>Mediastinal LN, pleural effusion, brain	EBRT<br><br>WBRT<br><br>WBRT	Vertebra T2, T3<br><br>Brain<br><br>Brain	Nivolumab<br><br>Atezolimumab<br><br>Pembrolizumab	Partial regression of adrenal metastases lasted for 10 months<br><br>Partial regression: brain met.; complete remission: lung, mediastinal masses<br><br>Reduction: lung mass, pleural effusion
Tubin S et al. [18] 2019	Clinical trial PCT	NSCLC (20)	IIIB/IV		SBRT-PATHY	Hypoxic tumor cells	no	AE observed in 45% (9/20) of patients
Bozorgmehr F et al. [19] 2019	Clinical trial PCT	NSCLC (130)	IV		Hypofractionated RT	All non-cerebral/non-pulmonary/non-internal organ met.	Nivolumab	

Liu X et al. [20] 2019	Case report	Case 1: ICC	IVC	Hepatic hilar and retroperitoneal LN	SBRT	Right hepatic lobe	Nivolumab	The sum of the metastatic lesions decreased by 40,9%
		Case 2: ICC	IIIA	Hepatic hilar and retroperitoneal LN	SBRT	Right hepatic lobe	Pembrolizumab	The sum of the metastatic lesions decreased by 86,3%
		Case 3: ICC	IIIB	Hepatic hilar and retroperitoneal LN	SBRT	Left hepatic lobe and left retroperitoneal LN	Pembrolizumab	CR lasted for 11 months
Tuyaerts S et al. [21] 2019	Clinical trial PCT	Cervical, endometrial carcinoma or uterine sarcoma (43)	Advanced		Hypofractionated RT		pembrolizumab	
Kuroda A et al. [22] 2019	Case report	Lung adenocarcinoma (1)	Metastatic	Supraclavicular, mediastinal and hilar LN, Lung	EBRT	Mediastinum	no	Disappearance of the lung metastases
Sundahl N et al. [23] 2019	Clinical trial	melanoma (20)	metastatic		SBRT	The largest lesion	Nivolumab	ORR 2 of 45% with 3 CR and 6 PR
Lin X et al. [24] 2019	Case report	Lung adenocarcinoma (1)	IV	Mediastinal LN, lung, pleural effusion, brain	SRT	Brain	Atezolimumab	Reduction: pleural effusion, lung, mediastinal LN lesions
Yaguchi D et al. [25] 2019	Case report	Pulmonary pleomorphic carcinoma (1)	Metastatic	Pleura, bone, brain	EBRT	Femur	Nivolumab	Near CR
Garcìa JR et al. [26] 2019	Case report	Lung adenocarcinoma (1)	metastatic	C6 vertebra	EBRT	C6	no	Reduction of the primary tumor
Gounder et al. [27] 2019	Case report	Sacral chordoma (1)	Metastatic	lung	EBRT	sacral mass	Tazemetostat+ nivolumab-ipilimumab	Regression of all lung nodules for 2 years
Mota JM et al. [28] 2019	Case report	Adrenocortical carcinoma (1)	metastatic	Liver, lung, pleura	RFA-SBRT	Liver, Lung-lung	Pembrolizumab	CR

Grimaux X et al. [29] 2019	Case report	RCC (1)	Metastatic	lung, costal	EBRT	costal	Nivolumab+rituximab	Disappearance of the lung metastases
Bonanno L et al. [30] 2019	Case report	SCLC (1)	Metastatic	Liver, peripancreatic LN	SBRT	Peripancreatic LN	Nivolumab	Reduction: hepatic lesion, the primary tumor
Formenti SC et al. [31] 2018	Clinical trial PCT	NSCLC (39)	metastatic		IGRT or IMRT		Ipilimumab	Abscopal response in 18% (7/39) of patients: 2 CR and 5 PR
Chuang CH et al. [32] 2018	Case report	Colorectal adenocarcinoma (1)	Metastatic	Liver, lung, brain	WBRT	Brain	no	Regression of lung mass
Chino F et al. [33] 2018	Case report	HCC+NSCLC (1)			SBRT	Lung tumor	no	Complete regression of Hepatic lesions
Britschgi C et al. [34] 2018	Case report	NSCLC (1)	cT1a pN3 cM0 (IIIB)	Abdominal LN	SBRT	2 of 3 LN	Nivolumab	CR
Xu MJ et al. [35] 2018	Case report	Merkel cell carcinoma (2)	Metastatic	Peripancreatic mass, adrenal gland, para-aortic LN	EBRT	Adrenal gland, para-aortic LN	Pembrolizumab	CR lasted for 17 months
Wight JC et al. [36] 2018	Case report	HL (1)		Lung, pleural effusion, LN	EBRT-inverted "Y" RT	Axillary LN-Infradiaphragmatic site	Nivolumab	CR in almost all areas
Rodrìguez-Ruiz ME et al. [37] 2018	Clinical trial PCT	15	Advanced		SABR	Selected tumor lesions	Hiltonol (poly-ICLC)	
Formenti SC et al. [38] 2018	Clinical trial PCT	Breast cancer 23)	Metastatic		Focal radiotherapy	Metastatic site	Fresolimumab	Response limited to 3 stable diseases
Deipolyi AR et al. [39] 2018	Case report	Breast cancer (1)	Metastatic	LN, lung, bone, brain, liver	Radioembolization	Liver	Pembrolizumab/utomilumab - Paclitaxel/trastuzumab - MEDI6469	CR
Leung HWC et al. [40] 2017	Case report	Breast cancer (1)	cT4N3bM1 (IV)	T8, axillary LN	EBT	Breast tumor, axillary LN	no	CR
Poon DMC et al. [41] 2017	Case report	Prostate adenocarcinoma (1)	Metastatic	bone, LN	Radium-223 - SBRT	C2, T6, S1, humerus	Denosumab	Significant regression of all lesions
Kwee SA et al. [42] 2017	Case report	CPRC (2)	Metastatic	LN, bone	223-Ra-Dichloride		no	Resolution of bone disease

Komatsu T et al. [43] 2017	Case report	Lung adenocarcinoma (1)	Metastatic	Liver, intrapulmonary		Liver	Nivolumab	Reduction: the primary tumor, intrapulmonary metastases
Tubin S et al. [44] 2017	Case report	Case 1: Lung adenocarcinoma<br><br>Case 2: SCC of the ear<br><br>Case 3: lung SCC	 <br>Metastatic<br><br>Metastatic	 <br>Neck dermal, lung, vertebra<br><br>Mediastinal LN, controlateral lung	EBRT<br><br>EBRT<br><br>EBRT	Hypoxic tumor segment<br><br>Hypoxic tumor segment<br><br>Hypoxic tumor segment	No<br><br>No<br><br>no	70% regression of the lesion<br><br>Complete regression of the primary tumor<br><br>Reduction: the primary tumor; disappearance: mediastinal LN
Xie G et al. [45] 2017	Case report	RCC (1)	T2a N1 M1 (IV)	Mediastinal, retroperitoneal, pelvis, cervical LN	SBRT	Para-aortic LN	Pembrolizumab	Systemic CR
Hidaka Y et al. [46] 2017	Case report	DLBCL (1)	Metastatic	skin	Topical RT	Buttock, arm	no	Disappearance of all skin lesions lasted for 6 months
LaPlant Q et al. [47] 2017	Case report	RCC (1)	Metastatic	LN, lung, sacrum, coccyx, pelvis	DP-SBRT	Sacrum mass	Ipilimumab+nivolumab	Reduction: sacral mass; disappearance: lung, LN metastases
Sharabi A et al. [48] 2017	Case report	Neuroendocrine cervical carcinoma (1)	Metastatic	Retroperitoneal, pelvic, para-aortic LN, liver	SBRT	Cervical mss	Nivolumab	Regression of 95% of all lesions
Shi F et al. [49] 2017	Case report	Pancreatic cancer (1)	Metastatic	Liver, pleura	EBRT	Pancreatic mass	GM-CSF subcutaneous	Reduction of the metastases
Foote M et al. [50] 2017	Clinical trial PCT	Melanoma (15)	Metastatic	In-transit metastases	Hypofractionated RT		PV-10	ORR: 86,6% (33,3% CR, 53,3% PR)
Gerber DE et al. [51] 2017	Clinical trial PCT	NSCLC	III		CRT or IMRT	Thoracic	Cislatin/etoposide+Nivlumab	
Cong Y et al. [52] 2017	Case report	Adenosquamous carcinoma of the chest wall (1)	Metastatic	lung	SABR	Paramediastinal and lingual lung foci	DC-CIK	Complete response
Desar IME et al. [53] 2016	Case report	DGCT (1)	Metastatic	Lung, hilar and mediastinal LN	EBRT	Hilar LN	Imatinib	Response of lung metastases lasted for 6 months

Levy A et al. [54] 2016	Clinical trial PCT	10	Metastatic		conformal 3D RT or intracranial stereotactic RT		Darvulumab	Objective response rate was 60% (CR 2/10 and PR 8/10)
Michot JM et al. [55] 2016	Case report	HL (1)		Supradiaphragmatic and subdiaphragmatic LN	EBRT	Hilar mediastinal LN	Pembrolizumab	Regression of the sum of volume of metastatic lesions
Van de Walle M et al. [56] 2016	Case report	RCC (1)	Metastatic	Portacaval and subcutaneous abdominal LN, lung, neck	IMRT	Neck mass	no	Complete regression: lung, sucutaneous abdominal LN metastases
Siva S et al. [57] 2016	Clinical trial	NSCLC (16)			3D-CRT	Primary tumor	Cisplatin/etoposide/carboplatin/paclitaxel	Unirradiated hair follicles: abscopal DNA damage response
Saba R et al. [58] 2016	Case report	Multiple myeloma (1)		Multiple osteolytic lesions, gastric and skull plasmacytoma	EBRT	Gastric Plasmacytoma	no	Regression: all nodular lesions; stability: bony lesions
Ghodadra A et al. [59] 2015	Case report	Lung SCC (1)	IV	Liver	Yttrium-90 radioembolization	via the right hepatic artery	no	Complete regression of the nontargeted lesion in segment IVB
Golden EB et al. [60] 2015	Clinical trial PCT	Solid tumor (41)	metastatic		EBRT	Metastatic site	GM-CSF injection	AE: in 11 (26,8%) of 41 enrolled patients
Okwan-Duodu D et al. [61] 2015	Case report	Melanoma (1)	metastatic	Brain, subcutaneous tissue, mesenteric, pelvic, retroperitoneal LN	WBRT	Brain	IL-2 and CTLA-4 therapy	CR lasted for over 3 years
Thallinger C et al. [62] 2015	Case report	Melanoma (1)	Metastatic	Lung, liver, kidney, adrenal glands, brain	WBRT	Brain	ipilimumab+temozolomide	Complete regression: brain, renal lesions; partial regression: liver, lung lesions
De La Cruz V et al. [63] 2014	Case report	Melanoma (1)	Metastatic	Lung, mediastinal LN, subcutaneous nodule	EBRT	Subcutaneous nodule	no	Regression: mediastinal LN, lung disease

Siva S et al. [64] 2013	Case report	Adenocarcinoma of lung (1)	IV	Adrenal gland, humeral head	EBRT + SABR	Left upper lobe tumor + right lower lobe	Carboplatin + paclitaxel	Regression: bone and adrenal lesions
Golden EB et al. [65] 2013	Case report	NSCLC (1)	IV	Liver, pelvis, TL spine, humerus, hilary/mediastinal LN	EBRT	Liver	ipilimumab	CR
Stamell Ef et al. [66] 2013	Case report	Melanoma (1)	IIIC	Skin of forehead, scalp, neck	EBRT	Primary tumor	no	CR lasted for 36 months
Sullivan RJ et al. [67] 2013	Case report	Melanoma (1)	Metastatic	Retroperitoneal, pelvic sidewall and inguinal LN, brain, bone	SRS	Brain	no	Regression: inguinal LN; bone lesion
Postow MA et al. [68] 2012	Case report	Melanoma (1)	Metastatic	Pleural-based paraspinal, hilar LN, spleen	EBRT	Paraspinal mass	Ipilimumab	Regression: treated and untreated metastases
Hiniker SM et al. [69] 2012	Pilot study PCT	Melanoma (1)	Metastatic	Liver, axillary LN	SBRT	Liver	ipilimumab	CR
Ishiyama H et al. [70] 2012	Case report	RCC (1)	pT2N0M1 (IV)	Adrenal gland, lung, bone, mediastinal and hilar LN, brain	SRS-SBRT	Brain -bone	no	Regression: lung, mediastinal disease; progression: brain metastases
Tubin et al. 2012	Case report	Medullary thyroid carcinoma (1)	pT3 pN1b (IVA)	Mediastinal LN	VMAT	Medistinal LN (level 4R)	no	32% reduction in the volume of mediastinal LN at level 6
Cotter SE et al. [71] 2011	Case report	Merkel cell carcinoma (1)	Metastatic	Skin	Brachytherapy	Skin	no	CR lasted for 25 months
Okuma K et al. [72] 2011	Case report	Hepatocellular carcinoma (1)	Metastatic	Lung, Mediastinal LN	EBRT		No	Reduction: mediastinal LN, lung lesions
Shueng PW et al. [73] 2009	Case report	Cholangiocarcinoma (1)	Metastatic	Vertebra tT8-L3, liver	HT	T8-T10	no	Acute radiation pneumonitis
Lakshmanagowda PB et al. [74] 2009	Case report	Chronic lymphocytic leukemia (1)		Axillary and cervical LN	EBRT	Axilla	no	Regression of neck LN
Isobe Y et al. [75] 2009	Case report	ENKL (1)		Submandibolar LN	EBRT	Periocular region	Chemotherapy CHOP	Eradication of all skin lesions, Regression of submandibolar LN

Nakanishi M et al. [76] 2008	Case report	Hepatocellular carcinoma (1)	Metastatic	Inferior vena cava (IVC)	EBRT	Right lobe tumor	Transcatheter arterial embolization for the posterior segment	CR of the right and left lobe tumors
Takaya M et al. [77] 2007	Case report	Uterine cervical carcinoma (1)	Advanced	Para-aortic LN	EBRT + Brachytherapy	Pelvis	no	CR
Wersäll P et al. [78] 2006	Case report	Case 1: RCC	Metastatic	Renal hilum LN, lung	SBRT	Renal mass	No	Complete regression of metastases
		Case 2: RCC	Metastatic	Lung	SBRT	Lung	No	CR: treated lung metastases; PR: untreated lung metastases
		Case 3: RCC	Metastatic	Lung	SBRT	Lung	No	CR: treated lung metastases; PR: untreated lung metastases
		Case 4: RCC	Metastatic	Kidney hilum and periaortic LN, liver	SBRT	Renal mass	No	CR
Nam SW et al. [79] 2005	Case report	Hepatocellular carcinoma (1)	Metastatic	Skull, 3-5 and 6th ribs, sternum	EBRT	Skull	No	Reduction: liver mass; CR: bone lesions
Ohba K et al. [80] 1998	Case report	Hepatocellular carcinoma (1)	Metastatic	Vertebra T2	EBRT	Vertebra	No	Reduction: liver mass
Sham RL et al. [81] 1995	Case report	Chronic lymphocytic leukemia (1)		Cervical LN, spleen	EBRT	neck, supraclavicular LN	no	Reduction: splenomegaly, WBC count
Braun MP and Maziarz RT 1994	Case report	HL (1)	4B	Spleen, liver, pulmonary, pleura, L5	EBRT	Lombosacral bone	no	Reduction of colony formation by mobilized stem cells
MacManus MP et al. [82] 1994	Case report	Renal cell carcinoma (RCC) (1)	Metastatic	Paratracheal and subcarinal LN, lung	EBRT	Renal mass	no	Reduction: paratracheal LN; regression: lung disease lasted for 9 months

Rees GJ et al. [83] 1983	Case report	Case 1: Adenocarcinoma of esophagus<br><br>Case 2: Adenocarcinoma of lung	Metastatic<br><br>Metastatic	Lung<br><br>Forehead, chest wall, deltoid muscle	EBRT<br><br>EBRT	Esophagus<br><br>Mediastinum and lower left lung	No<br><br>No	Regression: primary and lung disease lasted for 14 months<br><br>Regression: forehead and deltoid lesions lasted for 3 weeks
Rees GJ et al. [84] 1981	Retrospective survey	Case 1: Mixed-cellularity HL<br><br>Case 2: LPHL<br><br>Case 3: HL<br><br>Case 4: Mixed-cellularity HL<br><br>Case 5: Follicular lymphoma<br><br>Case 6: Follicular lymphoma<br><br>Case 7: Lymphosarcoma<br><br>Case 8: NHL<br><br>Case 9: Reticulum-cell sarcoma<br><br>Case 10: Follicular lymphoma	III<br><br>III<br><br>III<br><br>III		EBRT<br><br>EBRT<br><br>EBRT<br><br>EBRT<br><br>EBRT<br><br>EBRT<br><br>EBRT<br><br>EBRT<br><br>EBRT<br><br>EBRT	Mantle<br><br>Mantle<br><br>Para-aortic and pelvic LN<br><br>Mantle<br><br>Abdomen<br><br>Right axilla, chest wall<br><br>Right groin<br><br>Right breast, pelvis<br><br>Para-aortic and pelvic LN<br><br>Neck, supraclavicular aerea	no<br><br>no<br><br>no<br><br>no<br><br>no<br><br>no<br><br>no<br><br>no<br><br>no<br><br>no	Reduction: pelvic LN<br><br>Reduction: pelvic, para-aortic LN<br><br>Reduction: axillary LN lasted for 3 months<br><br>Reduction: para-aortic LN<br><br>Regression: pleural effusion lasted for 6 months<br><br>Regression: left axillary LN, spleen lesions<br><br>Regression: right neck LN lasted for 6 months<br><br>Regression: mediastinal mass<br><br>Regression: right axillary LN lasted for 6 months<br><br>Regression: bilateral axillary LN

Robins H I et al. 1981	Case report	Histiocytic lymphoma (1)	IV B	Right and Left kidneys	EBRT	Right kidney	Chemotherapy BACOP	24% reduction: the maximal axis of the rigth kidney;12,6% reduction: the left
Fairlamb DJ et al. [85] 1981	Case report	Clear cell carcinoma (1)	Metastatic	Lung, hila, pubic bone	EBRT	Groin mass	no	Regression: lung deposits
Antoniades J et al. [86] 1977	Case report	Case 1: lymphocytic lymphoma<br><br>Case 2: Lumphocytic lymphoma	IV<br><br>III	Axillary, supraclavicular, periaortic and pelvic LN, lung<br><br>Axillary, periaortic and pelvic LN, spleen	EBRT<br><br>EBRT	LN above diaphram, Waldeyer's ring, lung<br><br>LN above diaphram, Waldeyer's ring	No<br><br>No	LN regressed to <40%<br><br>LN regressed to <60%
Kurlander R et al. [87] 1975	Case report	Chronic lymphocytic leukemia (1)		Cervical, axillary and inguinal LN, spleen	EBRT	Spleen	no	Hyperkalemia attributed to the necrosis of leukemic cells
Kingsley D P [88] 1975	Case report	Melanoma (1)	Metastatic	Inguinal, iliac, para-aortic LN	Fast neutrons	Inguinal region	no	Complete regression of LN diseases
Ehlers G et al. [89] 1973	Case report	Papillary adenocarcinoma of unknown origin (1)	Metastatic	Supraclavicular LN, axilla	EBRT	Neck, supraclavicular area	no	Regression: supraclavicular, axillary lesions
Nobler M P [90] 1969	Case report	Follicular lymphoma (1)	IV	Cervical, axillary, inguinal, femoral, pelvic and para-aortic LN, liver, spleen	EBRT	Para-aortic, pelvic, femoral LN	no	CR

ACR= Abscopal disease control rate; AE= Abscopal effect; ARR= abscopal response rate; CR= complete response; CRC= colorectal cancer; CRT= 3D-conformal radiation therapy; DGCT= diffuse-type giant cell tumor; DP-SBRT= dose-painting SBRT; EBRT= external beam radiation therapy; EBT= electron beam therapy; ENKL= extranodal NK/T cell lymphoma, nasal type; HT= helical tomotherapy; HNSCC= head and neck squamous cell carcinoma; HT= helical tomotherapy; ICC= intrahepatic cholangiocarcinoma; IGRT= image-guided radiotherapy; IMRT= intensity modulated radiotherapy; LCNEC= Large-cell neuroendocrine carcinoma; LPHL= Lymphocyte predominant Hodgkin lymphoma; NHL= non-Hodgkin lymphoma; NSCLC= Non-small-cell lung carcinoma; OOR= overall objective response; ORR= overall response rate; PR= partial response; RCC= Renal cell carcinoma; RFA= radiofrequency ablation; SABR= stereotactic ablative body radiotherapy; SBRT= Stereotactic body radiation therapy; SBRT-PATHY= SBRT targeting partial tumor hypoxic; SCC= squamous cell carcinoma; SRS= Stereotactic radiosurgery; SRT= stereotactic radiotherapy; VMAT= volumetric modulated arc therapy; WBRT= Whole-brain radiotherapy

Bibliografia

1. Ye, H.; Pang, H.; Shi, X.; Ren, P.; Huang, S.; Yu, H.; Wu, J.; Lin, S. Nivolumab and Hypofractionated Radiotherapy in Patients With Advanced Lung Cancer: ABSCOPAL-1 Clinical Trial. *Front Oncol* **2021**, *11*, 657024, doi:10.3389/fonc.2021.657024.
2. Bassanelli, M.; Ricciuti, B.; Giannarelli, D.; Cecere, F.L.; Roberto, M.; Giacinti, S.; Barucca, V.; Santarelli, M.; Ruggeri, E.M.; Marchetti, P.; et al. Systemic effect of radiotherapy before or after nivolumab in lung cancer: an observational, retrospective, multicenter study. *Tumori* **2021**, 3008916211004733, doi:10.1177/03008916211004733.
3. Maity, A.; Mick, R.; Rengan, R.; Mitchell, T.C.; Amaravadi, R.K.; Schuchter, L.M.; Pryma, D.A.; Patsch, D.M.; Maity, A.P.; Minn, A.J.; et al. A stratified phase I dose escalation trial of hypofractionated radiotherapy followed by ipilimumab in metastatic melanoma: long-term follow-up and final outcomes. *Oncoimmunology* **2021**, *10*, 1863631, doi:10.1080/2162402X.2020.1863631.
4. Mattes, M.D.; Eubank, T.D.; Almubarak, M.; Wen, S.; Marano, G.D.; Jacobson, G.M.; Ma, P.C. A Prospective Trial Evaluating the Safety and Systemic Response From the Concurrent Use of Radiation Therapy with Checkpoint Inhibitor Immunotherapy in Metastatic Non-Small Cell Lung Cancer. *Clin Lung Cancer* **2021**, doi:10.1016/j.cllc.2021.01.012.
5. Li, D.; Zhu, W.; Zhou, J.; Peng, M.; Geng, Q.; Pu, X.; Wang, M.; Jiang, H. Hypofractionated Low-Dose Radiotherapy Combined with Immune Checkpoint Inhibition in Metastatic Solid Tumors. *Onco Targets Ther* **2021**, *14*, 773-783, doi:10.2147/OTT.S289937.
6. Kazandjian, D.; Dew, A.; Hill, E.; Ramirez, E.G.; Morrison, C.; Mena, E.; Lindenberg, L.; Yuan, C.; Maric, I.; Wang, H.W.; et al. Avelumab, a PD-L1 Inhibitor, in Combination with Hypofractionated Radiotherapy and the Abscopal Effect in Relapsed Refractory Multiple Myeloma. *Oncologist* **2021**, *26*, 288-e541, doi:10.1002/onco.13712.
7. Margulis, V.; Freifeld, Y.; Pop, L.M.; Manna, S.; Kapur, P.; Pedrosa, I.; Christie, A.; Mohamad, O.; Mannala, S.; Singla, N.; et al. Neoadjuvant SABR for Renal Cell Carcinoma Inferior Vena Cava Tumor Thrombus-Safety Lead-in Results of a Phase 2 Trial. *Int J Radiat Oncol Biol Phys* **2021**, *110*, 1135-1142, doi:10.1016/j.ijrobp.2021.01.054.
8. Segal, N.H.; Cercek, A.; Ku, G.; Wu, A.J.; Rimner, A.; Khalil, D.N.; Reidy-Lagunes, D.; Cuaron, J.; Yang, T.J.; Weiser, M.R.; et al. Phase II Single-arm Study of Durvalumab and Tremelimumab with Concurrent Radiotherapy in Patients with Mismatch Repair-proficient Metastatic Colorectal Cancer. *Clin Cancer Res* **2021**, *27*, 2200-2208, doi:10.1158/1078-0432.CCR-20-2474.
9. Theelen, W.; Chen, D.; Verma, V.; Hobbs, B.P.; Peulen, H.M.U.; Aerts, J.; Bahce, I.; Niemeijer, A.L.N.; Chang, J.Y.; de Groot, P.M.; et al. Pembrolizumab with or without radiotherapy for metastatic non-small-cell lung cancer: a pooled analysis of two randomised trials. *Lancet Respir Med* **2021**, *9*, 467-475, doi:10.1016/S2213-2600(20)30391-X.
10. Singh, S.A.; McDermott, D.M.; Mattes, M.D. Impact of Systemic Therapy Type and Timing on Intracranial Tumor Control in Patients with Brain Metastasis from Non-Small-Cell Lung Cancer Treated With Stereotactic Radiosurgery. *World Neurosurg* **2020**, *144*, e813-e823, doi:10.1016/j.wneu.2020.09.082.
11. Lieverse, R.I.Y.; Van Limbergen, E.J.; Oberije, C.J.G.; Troost, E.G.C.; Hadrup, S.R.; Dingemans, A.C.; Hendriks, L.E.L.; Eckert, F.; Hiley, C.; Dooms, C.; et al. Stereotactic ablative body radiotherapy (SABR) combined with immunotherapy (L19-IL2) versus standard of care in stage IV NSCLC patients, ImmunoSABR: a multicentre, randomised controlled open-label phase II trial. *BMC Cancer* **2020**, *20*, 557, doi:10.1186/s12885-020-07055-1.
12. Funck-Brentano, E.; Baghad, B.; Fort, M.; Aouidad, I.; Roger, A.; Beauchet, A.; Otmezguine, Y.; Blom, A.; Longvert, C.; Boru, B.; et al. Efficacy of late concurrent hypofractionated radiotherapy in advanced melanoma patients failing anti-PD-1 monotherapy. *Int J Cancer* **2020**, *147*, 1707-1714, doi:10.1002/ijc.32934.
13. Yano, M.; Aso, S.; Sato, M.; Aoyagi, Y.; Matsumoto, H.; Nasu, K. Pembrolizumab and Radiotherapy for Platinum-refractory Recurrent Uterine Carcinosarcoma With an Abscopal Effect: A Case Report. *Anticancer Res* **2020**, *40*, 4131-4135, doi:10.21873/anticanres.14412.
14. Watanabe, T.; Firat, E.; Scholber, J.; Gaedicke, S.; Heinrich, C.; Luo, R.; Ehrat, N.; Multhoff, G.; Schmitt-Graeff, A.; Grosu, A.L.; et al. Deep abscopal response to radiotherapy and anti-PD-1 in an oligometastatic melanoma patient with unfavorable pretreatment immune signature. *Cancer Immunol Immunother* **2020**, *69*, 1823-1832, doi:10.1007/s00262-020-02587-8.
15. Ellerin, B.E.; Demandante, C.G.N.; Martins, J.T. Pure abscopal effect of radiotherapy in a salivary gland carcinoma: Case report, literature review, and a search for new approaches. *Cancer Radiother* **2020**, *24*, 226-246, doi:10.1016/j.canrad.2020.01.001.
16. Choi, J.S.; Sansoni, E.R.; Lovin, B.D.; Lindquist, N.R.; Phan, J.; Mayo, L.L.; Ferrarotto, R.; Su, S.Y. Abscopal Effect Following Immunotherapy and Combined Stereotactic Body Radiation Therapy in Recurrent Metastatic Head and Neck Squamous Cell Carcinoma: A Report of Two Cases and Literature Review. *Ann Otol Rhinol Laryngol* **2020**, *129*, 517-522, doi:10.1177/0003489419896602.
17. Garelli, E.; Rittmeyer, A.; Putora, P.M.; Glatzer, M.; Dressel, R.; Andreas, S. Abscopal effect in lung cancer: three case reports and a concise review. *Immunotherapy* **2019**, *11*, 1445-1461, doi:10.2217/imt-2019-0105.
18. Tubin, S.; Khan, M.K.; Salerno, G.; Mourad, W.F.; Yan, W.; Jeremic, B. Mono-institutional phase 2 study of innovative Stereotactic Body RadioTherapy targeting PArtial Tumor HYpoxic (SBRT-PATHY) clonogenic cells in unresectable bulky non-small cell lung cancer: profound non-targeted effects by sparing peri-tumoral immune microenvironment. *Radiat Oncol* **2019**, *14*, 212, doi:10.1186/s13014-019-1410-1.
19. Bozorgmehr, F.; Hommertgen, A.; Krisam, J.; Lasitschka, F.; Kuon, J.; Maenz, M.; Huber, P.E.; Konig, L.; Kieser, M.; Debus, J.; et al. Fostering efficacy of anti-PD-1-treatment: Nivolumab plus radiotherapy in advanced non-small cell lung cancer - study protocol of the FORCE trial. *BMC Cancer* **2019**, *19*, 1074, doi:10.1186/s12885-019-6205-0.
20. Liu, X.; Yao, J.; Song, L.; Zhang, S.; Huang, T.; Li, Y. Local and abscopal responses in advanced intrahepatic cholangiocarcinoma with low TMB, MSS, pMMR and negative PD-L1 expression following combined therapy of SBRT with PD-1 blockade. *J Immunother Cancer* **2019**, *7*, 204, doi:10.1186/s40425-019-0692-z.
21. Tuyaerts, S.; Van Nuffel, A.M.T.; Naert, E.; Van Dam, P.A.; Vuylsteke, P.; De Caluwe, A.; Aspeslagh, S.; Dirix, P.; Lippens, L.; De Jaeghere, E.; et al. PRIMMO study protocol: a phase II study combining PD-1 blockade, radiation and immunomodulation to tackle cervical and uterine cancer. *BMC Cancer* **2019**, *19*, 506, doi:10.1186/s12885-019-5676-3.

22. Kuroda, A.; Tabuchi, T.; Iwami, E.; Sasahara, K.; Matsuzaki, T.; Nakajima, T.; Tsutsumi, Y.; Eguchi, K.; Terashima, T. Abscopal effect of radiation on multiple lung metastases of lung adenocarcinoma: a case report. *BMC Cancer* **2019**, *19*, 336, doi:10.1186/s12885-019-5566-8.
23. Sundahl, N.; Seremet, T.; Van Dorpe, J.; Neyns, B.; Ferdinande, L.; Meireson, A.; Brochez, L.; Kruse, V.; Ost, P. Phase 2 Trial of Nivolumab Combined With Stereotactic Body Radiation Therapy in Patients With Metastatic or Locally Advanced Inoperable Melanoma. *Int J Radiat Oncol Biol Phys* **2019**, *104*, 828-835, doi:10.1016/j.ijrobp.2019.03.041.
24. Lin, X.; Lu, T.; Xie, Z.; Qin, Y.; Liu, M.; Xie, X.; Li, S.; Zhou, C. Extracranial abscopal effect induced by combining immunotherapy with brain radiotherapy in a patient with lung adenocarcinoma: A case report and literature review. *Thorac Cancer* **2019**, *10*, 1272-1275, doi:10.1111/1759-7714.13048.
25. Yaguchi, D.; Ichikawa, M.; Ito, M.; Okamoto, S.; Kimura, H.; Watanabe, K. Dramatic response to nivolumab after local radiotherapy in pulmonary pleomorphic carcinoma with rapid progressive post-surgical recurrence. *Thorac Cancer* **2019**, *10*, 1263-1266, doi:10.1111/1759-7714.13029.
26. Garcia, J.R.; Soler, M.; Alvarez-Moro, F.J.; Mourelo, S.; Ortiz, S.; Riera, E. (18)F-FDG PET/CT in the diagnosis of the abscopal effect. A case report. *Rev Esp Med Nucl Imagen Mol (Engl Ed)* **2019**, *38*, 330-331, doi:10.1016/j.remn.2018.12.006.
27. Gounder, M.M.; Zhu, G.; Roshal, L.; Lis, E.; Daigle, S.R.; Blakemore, S.J.; Michaud, N.R.; Hameed, M.; Hollmann, T.J. Immunologic Correlates of the Abscopal Effect in a SMARCB1/INI1-negative Poorly Differentiated Chordoma after EZH2 Inhibition and Radiotherapy. *Clin Cancer Res* **2019**, *25*, 2064-2071, doi:10.1158/1078-0432.CCR-18-3133.
28. Mota, J.M.; Sousa, L.G.; Braghiroli, M.I.; Siqueira, L.T.; Neto, J.E.B.; Chapchap, P.; Hoff, A.A.O.; Hoff, P.M. Pembrolizumab for metastatic adrenocortical carcinoma with high mutational burden: Two case reports. *Medicine (Baltimore)* **2018**, *97*, e13517, doi:10.1097/MD.0000000000013517.
29. Grimaux, X.; Delva, R.; Jadaud, E.; Croue, A. Nivolumab-induced bullous pemphigoid after radiotherapy and abscopal effect. *Australas J Dermatol* **2019**, *60*, e235-e236, doi:10.1111/ajd.12987.
30. Bonanno, L.; Pavan, A.; Attili, I.; Pasello, G.; Guarneri, V. Immunotherapy in SCLC: Exceptional Clinical Benefit and Abscopal Pneumonitis After Radiotherapy. *J Thorac Oncol* **2019**, *14*, e5-e7, doi:10.1016/j.jtho.2018.08.2033.
31. Formenti, S.C.; Rudqvist, N.P.; Golden, E.; Cooper, B.; Wennerberg, E.; Lhuillier, C.; Vanpouille-Box, C.; Friedman, K.; Ferrari de Andrade, L.; Wucherpfennig, K.W.; et al. Radiotherapy induces responses of lung cancer to CTLA-4 blockade. *Nat Med* **2018**, *24*, 1845-1851, doi:10.1038/s41591-018-0232-2.
32. Chuang, C.H.; Hsu, J.F.; Shen, Y.T.; Yang, C.J. Regression of a metastatic lung mass after receiving whole brain irradiation: Can the abscopal effect cross the blood-brain barrier? *Asia Pac J Clin Oncol* **2018**, *14*, e548-e550, doi:10.1111/ajco.13051.
33. Chino, F.; Pollis, K.E.; Choi, S.; Salama, J.K.; Palta, M. Stereotactic Body Radiation Therapy-Induced Abscopal Effect on Hepatocellular Carcinoma After Treatment for Lung Cancer: A Case Report. *Hepatology* **2018**, *68*, 1653-1655, doi:10.1002/hep.30100.
34. Britschgi, C.; Riesterer, O.; Burger, I.A.; Guckenberger, M.; Curioni-Fontecedro, A. Report of an abscopal effect induced by stereotactic body radiotherapy and nivolumab in a patient with metastatic non-small cell lung cancer. *Radiat Oncol* **2018**, *13*, 102, doi:10.1186/s13014-018-1049-3.
35. Xu, M.J.; Wu, S.; Daud, A.I.; Yu, S.S.; Yom, S.S. In-field and abscopal response after short-course radiation therapy in patients with metastatic Merkel cell carcinoma progressing on PD-1 checkpoint blockade: a case series. *J Immunother Cancer* **2018**, *6*, 43, doi:10.1186/s40425-018-0352-8.
36. Wight, J.C.; Hawkes, E.A.; Berlangieri, S.U.; Khor, R.; Grigg, A.P. An abscopal effect may augment PD-1 inhibition in refractory classical Hodgkin lymphoma. *Leuk Lymphoma* **2018**, *59*, 2749-2751, doi:10.1080/10428194.2018.1452217.
37. Rodriguez-Ruiz, M.E.; Perez-Gracia, J.L.; Rodriguez, I.; Alfaro, C.; Onate, C.; Perez, G.; Gil-Bazo, I.; Benito, A.; Inoges, S.; Lopez-Diaz de Cerio, A.; et al. Combined immunotherapy encompassing intratumoral poly-ICLC, dendritic-cell vaccination and radiotherapy in advanced cancer patients. *Ann Oncol* **2018**, *29*, 1312-1319, doi:10.1093/annonc/mdy089.
38. Formenti, S.C.; Lee, P.; Adams, S.; Goldberg, J.D.; Li, X.; Xie, M.W.; Ratikan, J.A.; Felix, C.; Hwang, L.; Faull, K.F.; et al. Focal Irradiation and Systemic TGFbeta Blockade in Metastatic Breast Cancer. *Clin Cancer Res* **2018**, *24*, 2493-2504, doi:10.1158/1078-0432.CCR-17-3322.
39. Deipolyi, A.R.; Bromberg, J.F.; Erinjeri, J.P.; Solomon, S.B.; Brody, L.A.; Riedl, C.C. Abscopal Effect after Radioembolization for Metastatic Breast Cancer in the Setting of Immunotherapy. *J Vasc Interv Radiol* **2018**, *29*, 432-433, doi:10.1016/j.jvir.2017.10.007.
40. Leung, H.W.; Wang, S.Y.; Jin-Jhih, H.; Chan, A.L. Abscopal effect of radiation on bone metastases of breast cancer: A case report. *Cancer Biol Ther* **2018**, *19*, 20-24, doi:10.1080/15384047.2017.1394545.
41. Poon, D.M.C.; Wong, K.C.W. Lymph Node Response in a Patient With Metastatic Castration-resistant Prostate Cancer Treated With Radium-223. *Clin Genitourin Cancer* **2018**, *16*, e397-e401, doi:10.1016/j.clgc.2017.10.024.
42. Kwee, S.A.; Lim, J.; Coel, M.N. Soft Tissue Response on 18F-Fluorocholine PET/CT in Metastatic Castrate-Resistant Prostate Cancer Treated With 223Ra-Dichloride: A Possible Abscopal Effect? *Clin Nucl Med* **2017**, *42*, 868-871, doi:10.1097/RLU.0000000000001807.
43. Komatsu, T.; Nakamura, K.; Kawase, A. Abscopal Effect of Nivolumab in a Patient with Primary Lung Cancer. *J Thorac Oncol* **2017**, *12*, e143-e144, doi:10.1016/j.jtho.2017.05.004.
44. Tubin, S.; Raunik, W. Hunting for abscopal and bystander effects: clinical exploitation of non-targeted effects induced by partial high-single-dose irradiation of the hypoxic tumour segment in oligometastatic patients. *Acta Oncol* **2017**, *56*, 1333-1339, doi:10.1080/0284186X.2017.1346385.
45. Xie, G.; Gu, D.; Zhang, L.; Chen, S.; Wu, D. A rapid and systemic complete response to stereotactic body radiation therapy and pembrolizumab in a patient with metastatic renal cell carcinoma. *Cancer Biol Ther* **2017**, *18*, 547-551, doi:10.1080/15384047.2017.1345389.

46. Hidaka, Y.; Takeichi, T.; Ishikawa, Y.; Kawamura, M.; Akiyama, M. Abscopal Effect of Local Irradiation Treatment for Diffuse Large B-cell Lymphoma. *Acta Derm Venereol* **2017**, *97*, 1140-1141, doi:10.2340/00015555-2729.
47. LaPlant, Q.; Deselm, C.; Lockney, N.A.; Hsieh, J.; Yamada, Y. Potential abscopal response to dual checkpoint blockade in RCC after reirradiation using dose-painting SBRT. *Pract Radiat Oncol* **2017**, *7*, 396-399, doi:10.1016/j.prro.2017.04.009.
48. Sharabi, A.; Haran-Ghera, N. Immune recovery after cyclophosphamide treatment in multiple myeloma: implication for maintenance immunotherapy. *Bone Marrow Res* **2011**, *2011*, 269519, doi:10.1155/2011/269519.
49. Shi, F.; Wang, X.; Teng, F.; Kong, L.; Yu, J. Abscopal effect of metastatic pancreatic cancer after local radiotherapy and granulocyte-macrophage colony-stimulating factor therapy. *Cancer Biol Ther* **2017**, *18*, 137-141, doi:10.1080/15384047.2016.1276133.
50. Foote, M.; Read, T.; Thomas, J.; Wagels, M.; Burmeister, B.; Smithers, B.M. Results of a phase II, open-label, non-comparative study of intralesional PV-10 followed by radiotherapy for the treatment of in-transit or metastatic melanoma. *J Surg Oncol* **2017**, *115*, 891-897, doi:10.1002/jso.24580.
51. Gerber, D.E.; Urbanic, J.J.; Langer, C.; Hu, C.; Chang, I.F.; Lu, B.; Movsas, B.; Jeraj, R.; Curran, W.J.; Bradley, J.D. Treatment Design and Rationale for a Randomized Trial of Cisplatin and Etoposide Plus Thoracic Radiotherapy Followed by Nivolumab or Placebo for Locally Advanced Non-Small-Cell Lung Cancer (RTOG 3505). *Clin Lung Cancer* **2017**, *18*, 333-339, doi:10.1016/j.cllc.2016.10.009.
52. Cong, Y.; Shen, G.; Wu, S.; Hao, R. Abscopal regression following SABR for non-small-cell-lung cancer: A case report. *Cancer Biol Ther* **2017**, *18*, 1-3, doi:10.1080/15384047.2016.1264541.
53. Desar, I.M.; Braam, P.M.; Kaal, S.E.; Gerritsen, W.R.; Oyen, W.J.; van der Graaf, W.T. Abscopal effect of radiotherapy in a patient with metastatic diffuse-type giant cell tumor. *Acta Oncol* **2016**, *55*, 1510-1512, doi:10.1080/0284186X.2016.1243805.
54. Levy, A.; Massard, C.; Soria, J.C.; Deutsch, E. Concurrent irradiation with the anti-programmed cell death ligand-1 immune checkpoint blocker durvalumab: Single centre subset analysis from a phase 1/2 trial. *Eur J Cancer* **2016**, *68*, 156-162, doi:10.1016/j.ejca.2016.09.013.
55. Michot, J.M.; Mazeron, R.; Dercle, L.; Ammari, S.; Canova, C.; Marabelle, A.; Rose, S.; Rubin, E.; Deutsch, E.; Soria, J.C.; et al. Abscopal effect in a Hodgkin lymphoma patient treated by an anti-programmed death 1 antibody. *Eur J Cancer* **2016**, *66*, 91-94, doi:10.1016/j.ejca.2016.06.017.
56. Van de Walle, M.; Demol, J.; Staelens, L.; Rottey, S. Abscopal effect in metastatic renal cell carcinoma. *Acta Clin Belg* **2017**, *72*, 245-249, doi:10.1080/17843286.2016.1201614.
57. Siva, S.; Lobachevsky, P.; MacManus, M.P.; Kron, T.; Moller, A.; Lobb, R.J.; Ventura, J.; Best, N.; Smith, J.; Ball, D.; et al. Radiotherapy for Non-Small Cell Lung Cancer Induces DNA Damage Response in Both Irradiated and Out-of-field Normal Tissues. *Clin Cancer Res* **2016**, *22*, 4817-4826, doi:10.1158/1078-0432.CCR-16-0138.
58. Saba, R.; Saleem, N.; Peace, D. Long-term survival consequent on the abscopal effect in a patient with multiple myeloma. *BMJ Case Rep* **2016**, *2016*, doi:10.1136/bcr-2016-215237.
59. Ghodadra, A.; Bhatt, S.; Camacho, J.C.; Kim, H.S. Abscopal Effects and Yttrium-90 Radioembolization. *Cardiovasc Intervent Radiol* **2016**, *39*, 1076-1080, doi:10.1007/s00270-015-1259-0.
60. Golden, E.B.; Chhabra, A.; Chachoua, A.; Adams, S.; Donach, M.; Fenton-Kerimian, M.; Friedman, K.; Ponzo, F.; Babb, J.S.; Goldberg, J.; et al. Local radiotherapy and granulocyte-macrophage colony-stimulating factor to generate abscopal responses in patients with metastatic solid tumours: a proof-of-principle trial. *Lancet Oncol* **2015**, *16*, 795-803, doi:10.1016/S1470-2045(15)00054-6.
61. Okwan-Duodu, D.; Pollack, B.P.; Lawson, D.; Khan, M.K. Role of radiation therapy as immune activator in the era of modern immunotherapy for metastatic malignant melanoma. *Am J Clin Oncol* **2015**, *38*, 119-125, doi:10.1097/COC.0b013e3182940dc3.
62. Thallinger, C.; Prager, G.; Ringl, H.; Zielinski, C. [Abscopal effect in the treatment of malignant melanoma]. *Hautarzt* **2015**, *66*, 545-548, doi:10.1007/s00105-014-3567-8.
63. de la Cruz, V.; Sanz, A.; Torrego, J.C.; Bruno Fiorini, A. [The strange abscopal effect]. *Rev Clin Esp (Barc)* **2014**, *214*, 170-171, doi:10.1016/j.rce.2013.12.005.
64. Siva, S.; Callahan, J.; MacManus, M.P.; Martin, O.; Hicks, R.J.; Ball, D.L. Abscopal [corrected] effects after conventional and stereotactic lung irradiation of non-small-cell lung cancer. *J Thorac Oncol* **2013**, *8*, e71-72, doi:10.1097/JTO.0b013e318292c55a.
65. Golden, E.B.; Demaria, S.; Schiff, P.B.; Chachoua, A.; Formenti, S.C. An abscopal response to radiation and ipilimumab in a patient with metastatic non-small cell lung cancer. *Cancer Immunol Res* **2013**, *1*, 365-372, doi:10.1158/2326-6066.CIR-13-0115.
66. Stamell, E.F.; Wolchok, J.D.; Gnjatic, S.; Lee, N.Y.; Brownell, I. The abscopal effect associated with a systemic anti-melanoma immune response. *Int J Radiat Oncol Biol Phys* **2013**, *85*, 293-295, doi:10.1016/j.ijrobp.2012.03.017.
67. Sullivan, R.J.; Lawrence, D.P.; Wargo, J.A.; Oh, K.S.; Gonzalez, R.G.; Piris, A. Case records of the Massachusetts General Hospital. Case 21-2013. A 68-year-old man with metastatic melanoma. *N Engl J Med* **2013**, *369*, 173-183, doi:10.1056/NEJMcpc1302332.
68. Postow, M.A.; Callahan, M.K.; Barker, C.A.; Yamada, Y.; Yuan, J.; Kitano, S.; Mu, Z.; Rasalan, T.; Adamow, M.; Ritter, E.; et al. Immunologic correlates of the abscopal effect in a patient with melanoma. *N Engl J Med* **2012**, *366*, 925-931, doi:10.1056/NEJMoa1112824.
69. Hiniker, S.M.; Chen, D.S.; Knox, S.J. Abscopal effect in a patient with melanoma. *N Engl J Med* **2012**, *366*, 2035; author reply 2035-2036, doi:10.1056/NEJMc1203984.
70. Ishiyama, H.; Teh, B.S.; Ren, H.; Chiang, S.; Tann, A.; Blanco, A.I.; Paulino, A.C.; Amato, R. Spontaneous regression of thoracic metastases while progression of brain metastases after stereotactic radiosurgery and stereotactic body radiotherapy for metastatic renal cell carcinoma: abscopal effect prevented by the blood-brain barrier? *Clin Genitourin Cancer* **2012**, *10*, 196-198, doi:10.1016/j.clgc.2012.01.004.
71. Cotter, S.E.; Dunn, G.P.; Collins, K.M.; Sahni, D.; Zukotynski, K.A.; Hansen, J.L.; O'Farrell, D.A.; Ng, A.K.; Devlin, P.M.; Wang, L.C. Abscopal effect in a patient with metastatic Merkel cell carcinoma following radiation therapy: potential role of induced antitumor immunity. *Arch Dermatol* **2011**, *147*, 870-872, doi:10.1001/archdermatol.2011.176.

72. Okuma, K.; Yamashita, H.; Niibe, Y.; Hayakawa, K.; Nakagawa, K. Abscopal effect of radiation on lung metastases of hepatocellular carcinoma: a case report. *J Med Case Rep* **2011**, *5*, 111, doi:10.1186/1752-1947-5-111.
73. Shueng, P.W.; Lin, S.C.; Chang, H.T.; Chong, N.S.; Chen, Y.J.; Wang, L.Y.; Hsieh, Y.P.; Hsieh, C.H. Toxicity risk of non-target organs at risk receiving low-dose radiation: case report. *Radiat Oncol* **2009**, *4*, 71, doi:10.1186/1748-717X-4-71.
74. Lakshmanagowda, P.B.; Viswanath, L.; Thimmaiah, N.; Dasappa, L.; Supe, S.S.; Kallur, P. Abscopal effect in a patient with chronic lymphocytic leukemia during radiation therapy: a case report. *Cases J* **2009**, *2*, 204, doi:10.1186/1757-1626-2-204.
75. Isobe, Y.; Aritaka, N.; Sasaki, M.; Oshimi, K.; Sugimoto, K. Spontaneous regression of natural killer cell lymphoma. *J Clin Pathol* **2009**, *62*, 647-650, doi:10.1136/jcp.2008.062976.
76. Nakanishi, M.; Chuma, M.; Hige, S.; Asaka, M. Abscopal effect on hepatocellular carcinoma. *Am J Gastroenterol* **2008**, *103*, 1320-1321, doi:10.1111/j.1572-0241.2007.01782_13.x.
77. Takaya, M.; Niibe, Y.; Tsunoda, S.; Jobo, T.; Imai, M.; Kotani, S.; Unno, N.; Hayakawa, K. Abscopal effect of radiation on toruliform para-aortic lymph node metastases of advanced uterine cervical carcinoma--a case report. *Anticancer Res* **2007**, *27*, 499-503.
78. Wersall, P.J.; Blomgren, H.; Pisa, P.; Lax, I.; Kalkner, K.M.; Svedman, C. Regression of non-irradiated metastases after extracranial stereotactic radiotherapy in metastatic renal cell carcinoma. *Acta Oncol* **2006**, *45*, 493-497, doi:10.1080/02841860600604611.
79. Nam, S.W.; Han, J.Y.; Kim, J.I.; Park, S.H.; Cho, S.H.; Han, N.I.; Yang, J.M.; Kim, J.K.; Choi, S.W.; Lee, Y.S.; et al. Spontaneous regression of a large hepatocellular carcinoma with skull metastasis. *J Gastroenterol Hepatol* **2005**, *20*, 488-492, doi:10.1111/j.1440-1746.2005.03243.x.
80. Ohba, K.; Omagari, K.; Nakamura, T.; Ikuno, N.; Saeki, S.; Matsuo, I.; Kinoshita, H.; Masuda, J.; Hazama, H.; Sakamoto, I.; et al. Abscopal regression of hepatocellular carcinoma after radiotherapy for bone metastasis. *Gut* **1998**, *43*, 575-577, doi:10.1136/gut.43.4.575.
81. Sham, R.L. The abscopal effect and chronic lymphocytic leukemia. *Am J Med* **1995**, *98*, 307-308, doi:10.1016/S0002-9343(99)80380-5.
82. MacManus, M.P.; Harte, R.J.; Stranex, S. Spontaneous regression of metastatic renal cell carcinoma following palliative irradiation of the primary tumour. *Ir J Med Sci* **1994**, *163*, 461-463, doi:10.1007/BF02940567.
83. Rees, G.J.; Ross, C.M. Abscopal regression following radiotherapy for adenocarcinoma. *Br J Radiol* **1983**, *56*, 63-66, doi:10.1259/0007-1285-56-661-63.
84. Rees, G.J. Abscopal regression in lymphoma: a mechanism in common with total body irradiation? *Clin Radiol* **1981**, *32*, 475-480, doi:10.1016/s0009-9260(81)80310-8.
85. Fairlamb, D.J. Spontaneous regression of metastases of renal cancer: A report of two cases including the first recorded regression following irradiation of a dominant metastasis and review of the world literature. *Cancer* **1981**, *47*, 2102-2106, doi:10.1002/1097-0142(19810415)47:8<2102::aid-cncr2820470833>3.0.co;2-k.
86. Antoniades, J.; Brady, L.W.; Lightfoot, D.A. Lymphangiographic demonstration of the abscopal effect in patients with malignant lymphomas. *Int J Radiat Oncol Biol Phys* **1977**, *2*, 141-147, doi:10.1016/0360-3016(77)90020-7.
87. Kurlander, R.; Stein, R.S.; Roth, D. Hyperkalemia complicating splenic irradiation of chronic lymphocytic leukemia. *Cancer* **1975**, *36*, 926-930, doi:10.1002/1097-0142(197509)36:3<926::aid-cncr2820360313>3.0.co;2-g.
88. Kingsley, D.P. An interesting case of possible abscopal effect in malignant melanoma. *Br J Radiol* **1975**, *48*, 863-866, doi:10.1259/0007-1285-48-574-863.
89. Ehlers, G.; Fridman, M. Abscopal effect of radiation in papillary adenocarcinoma. *Br J Radiol* **1973**, *46*, 220-222, doi:10.1259/0007-1285-46-543-220.
90. Nobler, M.P. The abscopal effect in malignant lymphoma and its relationship to lymphocyte circulation. *Radiology* **1969**, *93*, 410-412, doi:10.1148/93.2.410.
